# Identification, validation, and characterization of approved and investigational drugs interfering with the SARS‐CoV‐2 endoribonuclease Nsp15

**DOI:** 10.1002/pro.70156

**Published:** 2025-05-15

**Authors:** Spyros D. Chatziefthymiou, Maria Kuzikov, Sara Afandi, Greta Kovacs, Sukrit Srivastava, Andrea Zaliani, Andrey Gruzinov, Guillaume Pompidor, Michele Lunelli, Golam Rizvee Ahmed, Jörg Labahn, Johanna Hakanpää, Björn Windshügel, Michael Kolbe

**Affiliations:** ^1^ Photon Science, DESY Hamburg Germany; ^2^ Department for Structural Infection Biology Centre for Structural Systems Biology (CSSB) & Helmholtz‐Centre for Infection Research Hamburg Germany; ^3^ Fraunhofer Institute for Translational Medicine and Pharmacology ITMP, Discovery Research ScreeningPort Hamburg Germany; ^4^ School of Science, Constructor University Bremen Germany; ^5^ Forschungszentrum Jülich, Institute of Complex Systems (ICS‐6) Jülich Germany; ^6^ Faculty of Mathematics, Informatics and Natural Sciences Universität Hamburg Hamburg Germany

**Keywords:** drug screening, molecular docking, repurposing, SARS‐CoV‐2, uridine‐specific endoribonuclease Nsp15, x‐ray crystal structure

## Abstract

Since the emergence of SARS‐CoV‐2 at the end of 2019, the virus has caused significant global health and economic disruptions. Despite the rapid development of antiviral vaccines and some approved treatments such as remdesivir and paxlovid, effective antiviral pharmacological treatments for COVID‐19 patients remain limited. This study explores Nsp15, a 3′‐uridylate‐specific RNA endonuclease, which has a critical role in immune system evasion and hence in escaping the innate immune sensors. We conducted a comprehensive drug repurposing screen and identified 44 compounds that showed more than 55% inhibition of Nsp15 activity in a real‐time fluorescence assay. A validation pipeline was employed to exclude unspecific interactions, and dose–response assays confirmed 29 compounds with an IC_50_ below 10 μM. Structural studies, including molecular docking and x‐ray crystallography, revealed key interactions of identified inhibitors, such as TAS‐103 and YM‐155, with the Nsp15 active site and other critical regions. Our findings show that the identified compounds, particularly those retaining potency under different assay conditions, could serve as promising hits for developing Nsp15 inhibitors. Additionally, the study emphasizes the potential of combination therapies targeting multiple viral processes to enhance treatment efficacy and reduce the risk of drug resistance. This research contributes to the ongoing efforts to develop effective antiviral therapies for SARS‐CoV‐2 and possibly other coronaviruses.

## 1 INTRODUCTION

The severe acute respiratory syndrome coronavirus 2 (SARS‐CoV‐2) emerged at the end of 2019 (WHO, 2024). Since then, it has had a strong negative impact on health and economic systems worldwide and still causes hundreds of deaths daily (Faramarzi et al., 2024; WHO, 2024). The rapidly increasing knowledge about SARS‐CoV‐2 host manipulation has allowed the development of multiple approaches to contain the spread of infection, and in particular, the rapid development of antiviral vaccines was essential for pandemic control. However, there are still no effective antiviral pharmacological treatments available for COVID‐19 patients. Veklury (remdesivir) gained the broadest approval by regulatory authorities and is currently used in the EU, USA, Japan, and Australia (US FDA, 2020). The Food and Drug Administration (FDA) in 2021 and the European Medicines Agency (EMA) in 2022 approved Paxlovid® (combination of nirmatrelvir with ritonavir) for the treatment of COVID‐19 in the US and EU, respectively (European Medicines Agency, 2020; US FDA, 2020). In July 2021, the FDA issued the Emergency Use Authorization (EUA) for Eli Lilly's Janus kinase 1 and Janus kinase 2 (JAK1 and JAK2) inhibitor baricitinib (Olumiant®) as monotherapy, which is already approved for severe rheumatoid arthritis (Jorgensen et al., 2020; US FDA, 2022). Molnupiravir, a nucleoside analogue originally tested for inhibition of influenza and respiratory syncytial virus, was approved by the FDA for emergency use authorization for the treatment of mild‐to‐moderate COVID‐19. However, it was withdrawn rapidly due to safety issues (European Medicines Agency, 2023; Yoon et al., 2018).

Viral evolution often leads to a fast adaptation and eventually drug resistance, as shown from previous data, for example, on human immunodeficiency virus and hepatitis C virus, for which single drug therapies were often not effective (Mathis et al., 2011). Similar phenomena are also reported for SARS‐CoV‐2 as well (Abdelnabi et al., 2023; Iketani et al., 2023; Zhou et al., 2022). In contrast to this, therapies combining two or more antiviral drugs, like in HIV therapy, improve the treatment outcome by targeting multiple sites of the viral replication cycle, resulting in reduced viral load and mutation‐based escape (Foucquier & Guedj, 2015). Thus, further targets of the SARS‐CoV‐2 genome are under investigation as possible starting points for the development of new monotherapies or combination therapies.

One of the possible drug targets for SARS‐CoV2 is Nsp15, a 3′‐uridylate‐specific RNA endonuclease (NendoU) that plays a pivotal role in viral replication and transcription (Ivanov et al., 2004). Sequence analysis has shown that SARS‐CoV‐2 Nsp15 shares 88% and ~50% sequence identity with its orthologs from SARS‐CoV and MERS, respectively (Kim et al., 2020).

Structurally, SARS‐CoV‐2 Nsp15 forms a double‐ring‐shaped hexamer that consists of a dimer of trimers. All Nsp15 subunits in the hexamer can be occupied simultaneously by substrate molecules, whereas disruption of the hexamer significantly reduces the enzymatic activity (Deng & Baker, 2018; Kim et al., 2020). Each Nsp15 subunit is composed of three domains (Figure S1.). The N‐terminal domain is formed by an antiparallel *β*‐sheet (strands *β*1–*β*3) arranged around two *α*‐helices (*α*1 and *α*2) and is required for hexamer formation (Deng & Baker, 2018; Guarino et al., 2005; Horrell et al., 2022). The middle domain contains three *β*‐hairpins (*β*5–*β*6, *β*7–*β*8, and *β*12–*β*13), a mixed *β*‐sheet (*β*4, *β*9, *β*10, *β*11, *β*14, and *β*15), two α‐helices (*α*3 and *α*5), and one 3_10_ helix (*η*4). Finally, the C‐terminal catalytic EndoU domain is formed by two *β*‐sheets (*β*16–*β*18 and *β*19–*β*21) and five α‐helices (*α*6–*α*10) (Kim et al., 2020). The catalytic triad, found in a positively charged groove, is formed by a lysine (Lys290) and two histidine (His235 and His250) residues (Kim et al., 2020). The enzymatic activity is characterized by the formation of 2′‐3′‐cyclic phosphodiester and a 5′‐OH terminus, and it seems to be stimulated by Mn^2+^ (Bhardwaj et al., 2006; Huang et al., 2023; Ivanov et al., 2004). However, the metal binding site within Nsp15 has not been identified yet, and its importance for Nsp15 catalytic activity is under debate (Godoy et al., 2023). In the catalytic triad, His235 acts as an acid and His250 as a base (Figure S2). In addition, upstream residues Ser294 and Tyr343 have been shown to play an important role in the uridylate specificity of the enzyme (Huang et al., 2023; Kim et al., 2020). Due to its uridylate specificity, Nsp15 is capable of cleaving dsRNA, to a lesser extent ssRNA, but not DNA substrates (Ivanov et al., 2004). Residues of the C‐terminal domain are important for recognition by SARS‐CoV‐2 main protease (Mpro or 3CLpro) and thus for the efficient and controlled release of Nsp15 from the polyprotein (Bhardwaj et al., 2006).

Although Nsp15 has been initially proposed to be involved in virus replication, recent data showed that Nsp15‐deficient coronaviruses are able to replicate and form infectious particles. Its role in immune system evasion has been highlighted by its function in degrading dsRNA and hence in escaping the innate immune sensors, such as RIG‐I and MDA5 (Deng et al., 2017; Deng & Baker, 2018; Otter et al., 2024). It has also been shown to be a potent interferon antagonist by suppressing the production and signaling of IFN and inhibiting the nuclear localization of IRF3 (Yuen et al., 2020; Zhang et al., 2023).

Here, we report a drug repurposing approach coupled with structural and biophysical evaluation to identify approved and investigational drugs capable of inhibiting SARS‐CoV‐2 Nsp15 activity. By using a real‐time fluorescence assay, we were able to identify a series of novel Nsp15 inhibitors, one of which blocks the nucleotide‐binding pocket of the enzyme. By molecular docking, we were able to identify two more putative Nsp15 binding sites for small‐molecule ligands. Furthermore, we also identified a group of compounds possessing a 2,4‐diamino‐6,7‐dimethoxyquinazoline core, previously described as G9a/GLP histone methyltransferase inhibitors, that inhibit Nsp15.

## 2 RESULTS

**FIGURE 1:**
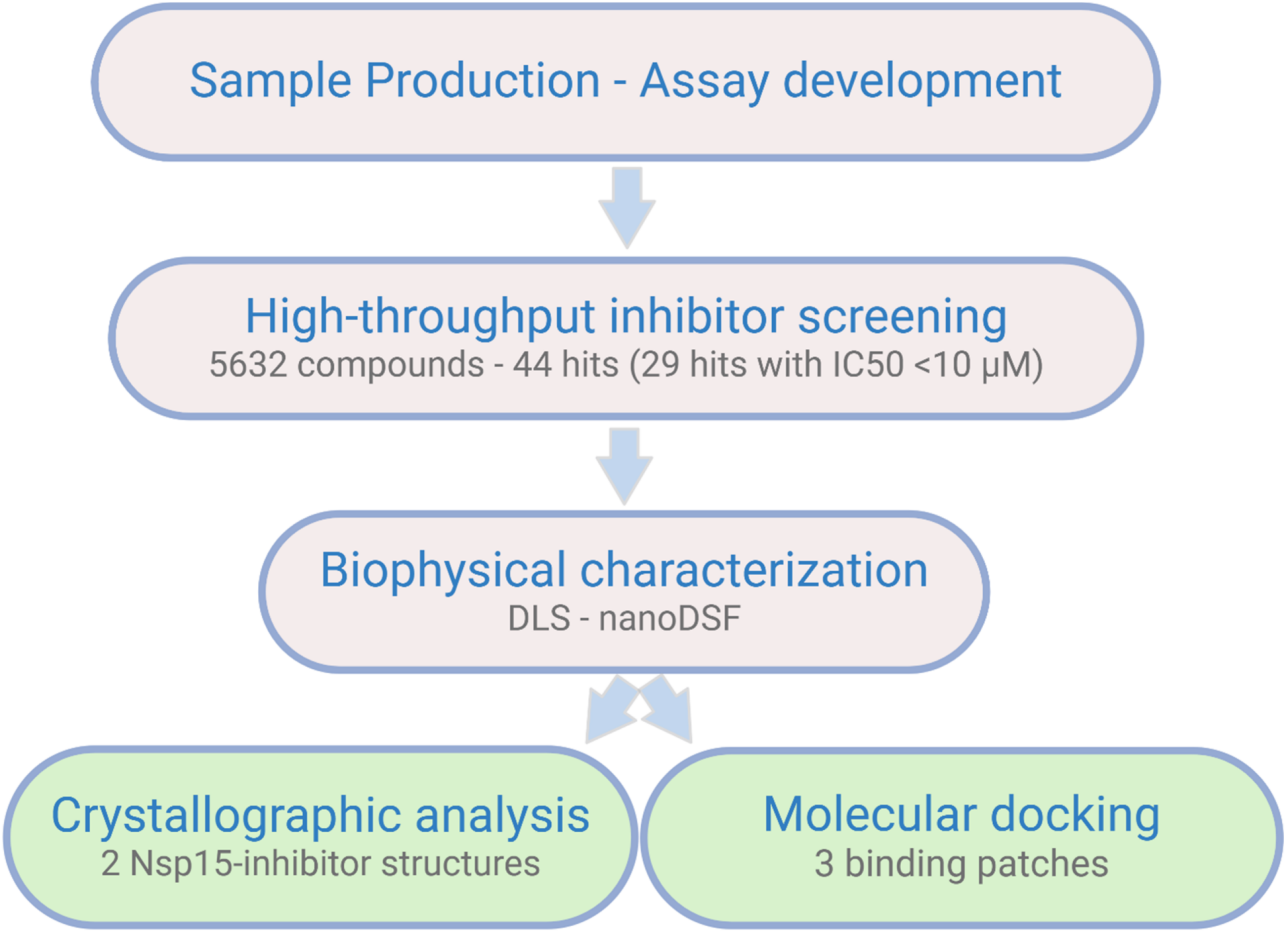
Schematic overview of the workflow followed in this study.

In this work, we adapted a biochemical test system for a high‐throughput compatible screening based on a recently reported real‐time fluorescence assay (Figure S3) (Kim et al., 2020). The identified inhibitors were further assessed by using a validation pipeline including structural determination and biophysical investigations of the enzyme–inhibitor interactions (Figure 1).

### 2.1 Characterization of Nsp15 enzymatic activity and assay adaptation

**FIGURE 2:**
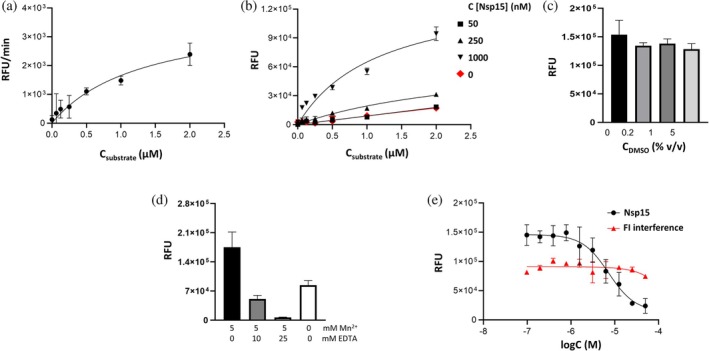
Characterization of Nsp15 enzymatic parameters. (a) Determination of *K*_m_. 1 μM Nsp15 was incubated with increasing substrate concentration and signal was detected every minute for a maximum of 5 min after substrate addition. (b) Titration of Nsp15. The enzyme was incubated in increasing concentrations, with increasing concentrations of the substrate or buffer (0 nM substrate). (c) Tolerance of Nsp15 enzymatic activity to increasing DMSO concentration in the assay, using 0–5 v/v % DMSO in assay. (d) Effect of EDTA addition and absence of Mn^2+^ in assay buffer on Nsp15 enzymatic activity. (e) Inhibition of Nsp15 using Congo red, calculated IC_50_ 7.5 μM. Each data point represents *n* = 3, error bars ±SD.

To optimize the activity assay for high‐throughput screening of new inhibitors, we determined key kinetic parameters of Nsp15 enzymatic activity (Figure 2). Using Nsp15 at 1 μM concentration, a *K*_m_ of 1.3 μM and a *V*_max_ of 3836 RFU/min were determined (Figure 2a). We obtained the best signal/background ratio (S/B) of 5.9 using 1 μM Nsp15 and 1 μM substrate (~*K*_m_) (Figure 2b). An increase in DMSO concentration up to 5 v/v % did not inhibit enzymatic activity (Figure 2c). Since the role of manganese on the enzyme activity is not fully understood yet and contradictory results have been reported for Mn^2+^ dependency (Godoy et al., 2023; Saramago et al., 2022), we monitored the Nsp15 activity upon addition of Mn^2+^ to the assay buffer. We observed an ~50% reduction of the fluorescence signal when Mn^2+^ was omitted from the assay buffer compared to the signal obtained using buffer supplemented with 5 mM Mn^2+^. Furthermore, the addition of 25 mM EDTA to the assay buffer for chelating manganese ions led to a reduction of the fluorescence signal to background level, indicating that this element is essential for the enzymatic activity of Nsp15 (Figure 2d).

We used the previously reported Nsp15 inhibitor Congo red to validate the optimized conditions (Ortiz‐Alcantara et al., 2010). Nsp15 was pre‐incubated with increasing concentrations of Congo red, followed by the addition of substrate. The resulting IC_50_ of 7.5 μM is in line with previously reported data for the inhibition potency of SARS‐CoV Nsp15 of 2.5 μM (Figure 2e) (Ortiz‐Alcantara et al., 2010). We did not observe any compound‐related interference with the generated fluorescence product.

### 2.2 Primary screen (PS)

**FIGURE 3:**
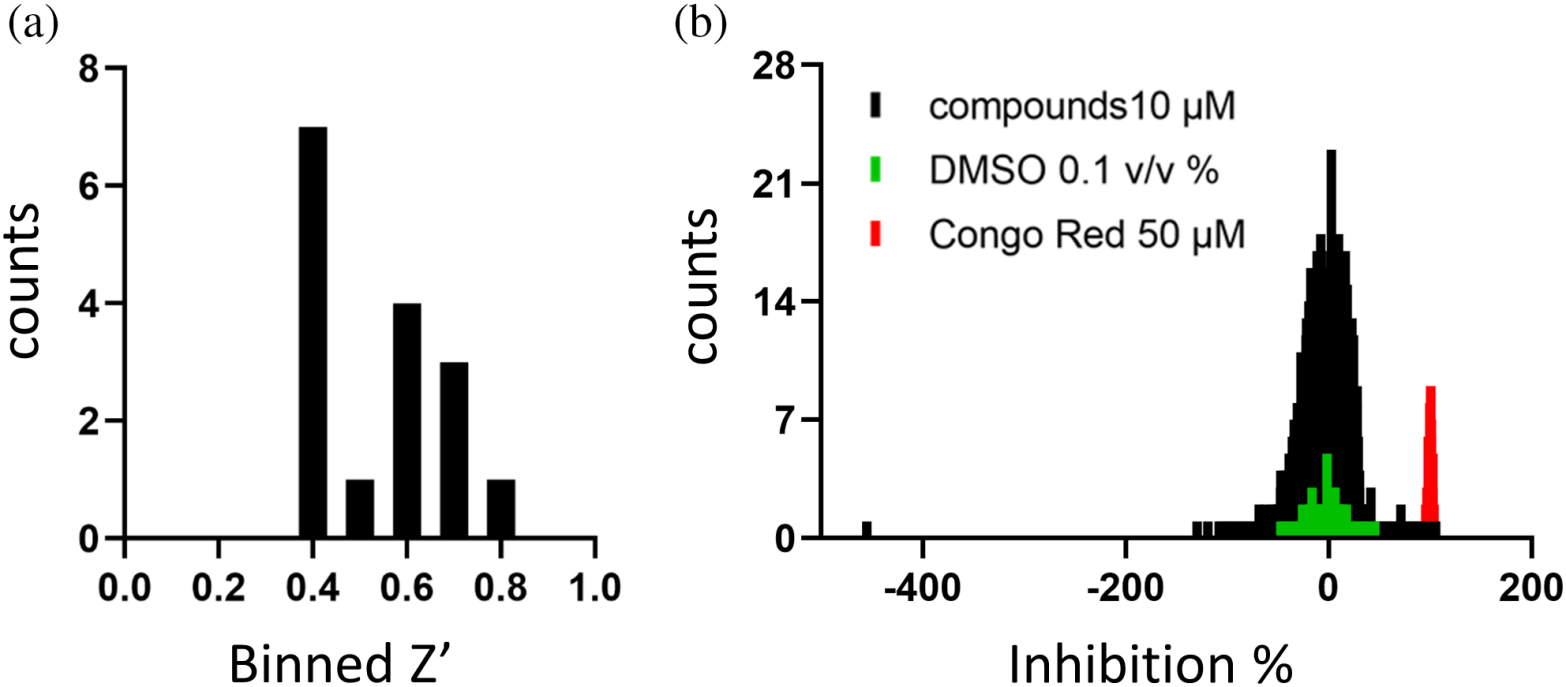
Primary screen (PS) and hit confirmation (HC) of the repurposing collection against SARS‐CoV‐2 Nsp15. (a) Frequency distribution of *Z*′ values calculated for screened assay plates. (b) Frequency distribution of calculated Nsp15 inhibition including the negative control/DMSO control and the positive control/100% inhibition (Congo red at 50 μM).

For the identification of potent Nsp15 inhibitors, we utilized the “Fraunhofer Repurposing Collection,” which comprises 5632 different approved and investigational drugs. This library has been assembled based on the design of the Broad Repurposing collection and includes 3400 clinically approved drugs across 600 indications, 1582 preclinical compounds, and compounds with varying degrees of validation (Corsello et al., 2017). The inhibitory effect of all screened compounds was tested on the basis of the Nsp15 enzymatic assay described above. Congo red was used as a positive control for inhibition, representing 100% inhibition. DMSO (0.1%) was used as solvent control, representing 0% inhibition. The fluorescence signal was detected 15, 30, and 45 min after substrate addition to allow a well‐based normalization. With the exception of one assay plate, all screened plates fulfilled the quality control of Z′ > 0.4, showing an average S/B ratio of 20.19 (with S = DMSO control; B = Congo red 50 μM) (Figure 3). In total, 44 compounds (0.78% hit rate) showed >55% inhibition of Nsp15 enzymatic activity and were selected for subsequent hit confirmation (HC). We confirmed 81.8% of the selected hits with more than 55% inhibition (Table S1). To determine if the observed inhibition was due to interference with the fluorophore, Nsp15 was incubated with the substrate for 1 h to let the assay run to completion, followed by addition of each confirmed hit. Two compounds, daunorubicin and novobiocin sodium, showed strong autofluorescence and thus were excluded from further characterization. Homidium bromide showed the highest quenching ratio of the fluorescence signal of 26.13% normalized to the DMSO control and was also excluded.

### 2.3 Hit profiling and biophysical characterization of Nsp15 inhibitor complexes

The 41 selected compounds of the primary screen were further tested in a dose‐dependent manner to determine the IC_50_ values. Finally, we found 29 compounds inhibiting the Nsp15 enzymatic activity with an IC_50_ value below 10 μM, with walrycin B, BVT‐948, and NSC‐663284 being the most potent inhibitors (Table 1).

**TABLE 1:** Characterization of SARS‐CoV‐2 Nsp15 inhibitors identified in the drug repurposing primary screen.

Inhibitor	PS inhibition % 10 μM (single point measurement)	IC_50_ + DTT (μM) (CI in μM)	IC_50_ + l‐Cys (μM) (CI in μM)	Main peak radius DLS (SD)	Polydispersity index (SD)	Δ*T*_m_°C nDSF
Octenidine dihydrochloride	98.6	3.8 (3.3–4.4)	4.4 (n.d.)	n.t	n.t	n.t.
Semapimod	106.9	0.6 (0.5–0.7)	1.0 (0.9–1.3)	n.t	n.t.	n.t.
UNC0646	75.3	1.4 (0.03–18.5)	0.7 (0.002–1.7)	5.32 (0.31)	0.11 (0.03)	0
Oritavancin (diphosphate)	86.7	2.5 (1.6–3.9)	1.2 (0.7–1.8)	5.19 (0.14)	0.28 (0.01)	0
Mitoxantrone hydrochloride	91.6	1.5 (0.8–3.0)	0.5 (n.d.–0.9)	4.85 ()	0.41 (−)	−0.2^a^
UNC0224	70.6	2.6 (0.02–89.4)	4.0 (n.d.–7.8)	5.48 (0.12)	0.06 (0.01)	−2.4
Epirubicin hydrochloride	70.9	3.4 (n.d.–15.2)	1.5 (0.007–3.0)	5.2 (0.21)	0.11 (0.03)	0.4
UNC0642	56.9	6.3 (n.d.)	3.3 (n.d.–10.2)	4.73 (−)	0.53 (−)	−5.2^a^
UNC0631	84.8	2.2 (0.03–53.3)	2.5 (1.9–6.6)	4.95 (0.17)	0.18 (0.16)	−0.1
GSK J4 HCl	75.8	5.5 (3.9–7.9)	3.8 (2.3–1274)	5.23 (0.17)	0.08 (0.03)	0.3
Tolonium chloride	72.0	1.7 (0.9–n.d.)	1.3 (0.2–2.4)	5.21 (0.03)	0.24 (0.13)	1.2
Doxorubicin	80.2	3.7 (0.2–34.8)	2.3 (1.3–4.7)	5.01 (0.28)	0.13 (0.08)	0.4
UNC0321	63.9	3.9 (n.d–19.6)	3.2 (n.d.–5.4)	5.29 (0.06)	0.37 (0.01)	−2.4
Pixantrone (dimaleate)	82.3	5.2 (0.06–42.8)	4.4 (1.6–n.d.)	4.96 (0.18)	0.21 (0.22)	−0.1
UNC0638	56.7	2.8 (0.09–505.0)	4.8 (3.3–7.6)	5.23 (0.28)	0.19 (0.09)	−1.1
Propidium‐iodide	83.1	0.9 (0.7–n.d.)	9.0 (4.2–12.5)	211.76	0.34 (0.21)	0.7
Nemorubicin	65.2	2.6 (n.d.)	1.3 (n.d.)	4.94 (0.05)	0.4 (0.07)	0.5^a^
Erdafitinib	57.2	3.2 (n.d.)	4.3 (n.d.–17.4)	5.19 (0.83)	0.26 (0.3)	−0.4
NSC‐663284	85.5	0.5 (n.d.–0.7)	n.t	4.85 (0.17)	0.42 (0.16)	1.8
IPA‐3	86.6	0.9 (0.8–1.1)	n.t	failed	failed	2
APTO‐253	61.1	7.8 (4.1–10.4)	n.t	4.48 ()	0.78 (−)	0.2
alphaLapachone	76.0	2.2 (0.09–17.3)	n.t	n.t	n.t	n.t.
Delavirdine (mesylate)	80.7	2.3 (0.05–21.5)	n.t	5.29 (0.32)	0.1 (0.11)	0.2
Walrycin B	86.2	<0.2 (n.d.)	n.t	5.29 (0.39)	0.44 (0.08)	3.2
EUK 134	70.9	3.3 (1.9–n.d.)	n.t	5.34 (0.17)	0.1 (0.11)	0.1
Elacridar	68.3	4.9 (0.005–n.d.)	n.t	6.05 (0.02)	0.11 (0.01)	2.3
YM‐155	74	1.2 (0.001–162.4)	n.t	5.26 (0.2)	0.1 (0.11)	0.1
BVT‐948	72.9	0.4 (n.d.–0.7)	n.t	5.36 (0.1)	0.05 (0.04)	3.7
TAS‐103 (dihydrochloride)	63.4	6.6 (3.9–8.4)	n.t	4.94 (0.09)	0.32 (0.05)	0.6

*Note*: Apo protein: main peak radius = 5.04 (0.21); *T*_m_ = 60.6 (0.1)°C; polydispersity index (SD) = 0.10 (0.01).

Abbreviations: CI, confidence interval; n.d., not defined/very wide concentration range; n.t., not tested.

^a^ For these ligands, there were unfolding events at lower temperatures.

Previously, we reported that in the presence of the reducing agent DTT, the compound activity can be affected if proteins contain solvent‐exposed cysteines and/or histidines, which are sensitive to oxidation (Kuzikov et al., 2021). Compounds possessing a quinone‐like moiety, for example, NSC‐663284, have been reported to act by generation of reactive oxygen species (ROS) (Gopinath et al., 2016; Proj et al., 2022). This mode of action was also reported in 2021 by Choi et al., who highlighted subgroups of small molecules that inhibit SARS‐CoV‐2 Nsp15 non‐specifically by ROS generation (Choi et al., 2021). We further observed that Nsp15 is highly sensitive to oxidation, resulting in a loss of enzymatic activity in the absence of a reducing agent. l‐Cysteine has a lower reducing potential compared to DTT while retaining Nsp15 activity (Figure S4) (Jocelyn, 1967; Zannini et al., 2017). For those reasons, we repeated the activity tests in buffer supplemented with 1 mM l‐cysteine for hits showing <10 μM IC_50_ in our previous assay. BVT‐948, YM‐155, delavirdine (mesylate), elacridar, alpha‐lapachone, walrycin B, TAS‐103 (dihydrochloride), and EUK 134 lost more than 50% of the inhibition potency in l‐cysteine buffer, indicating a possible redox‐dependent mechanism of action. Compounds that retained >55% inhibition in l‐cysteine buffer were re‐tested in dose–response in this less reducing environment (Table 1).

**FIGURE 4:**
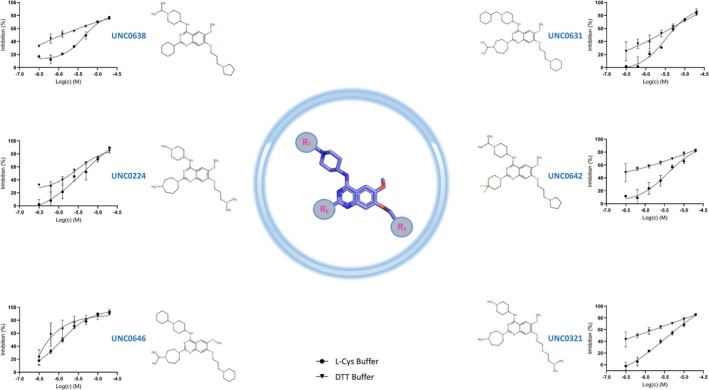
Structure of the found UNC compounds and their dose response curve series. Nsp15 was preincubated for 30 min with increasing concentrations of UNC‐compounds in the presence of 1 mM DTT or 1 mM l‐cysteine. Each data point represents *N* = 3, error bars ±SD.

Among the redox‐insensitive compounds that remained active in l‐cysteine buffer, we identified a hit series of 6,7‐dimethoxyquinazoline analogues previously reported to be histone methyltransferase G9a inhibitors (Figure 4) (Liu et al., 2009; Srimongkolpithak et al., 2014). UNC0646 showed the highest potency for Nsp15 inhibition with an IC_50_ of 1.36 and 0.73 μM in DTT and l‐cysteine buffer, respectively.

To address whether the inhibitors affect the oligomeric state of the enzyme, as well as its homogeneity and stability, we performed dynamic light scattering (DLS) and nano‐differential scanning fluorimetry (nanoDSF) analysis, using the inherent fluorescence of proteins. DLS measurements showed that, except for propidium iodide, none of the tested inhibitors significantly affected the hexameric Nsp15 structure, as indicated by the measured particle. Upon addition of the tested compounds, Nsp15 remained stable, with a *T*_m_ higher than 57°C (Table 1). Nevertheless, upon addition of the inhibitors, the samples showed a higher degree of heterogeneity than the ligand‐free Nsp15, as shown by the polydispersity index of these complexes, and in some cases, we observed unfolding events of low intensity at lower temperatures (Table 1).

### 2.4 Crystal structures of Nsp15 with the identified inhibitors

Following the inhibitor identification and the biophysical characterization of the Nsp15–inhibitor complexes, we attempted to structurally determine the binding mode of the identified inhibitors to Nsp15. First, the ligand‐free structure of Nsp15 was obtained, followed by co‐crystallization and soaking trials in the presence of the identified inhibitors. Apo‐protein crystals diffracted at a resolution of 2 Å or higher in the tested crystallization conditions. The Nsp15 structures obtained from the two crystallization conditions used in this work were isomorphous, with their asymmetric unit containing two Nsp15 molecules. The electron density map was of high quality and allowed us to trace the whole chain except the N‐terminal tag that could not be traced in any of the structures, and the last three C‐terminal residues that were not well resolved.

**FIGURE 5:**
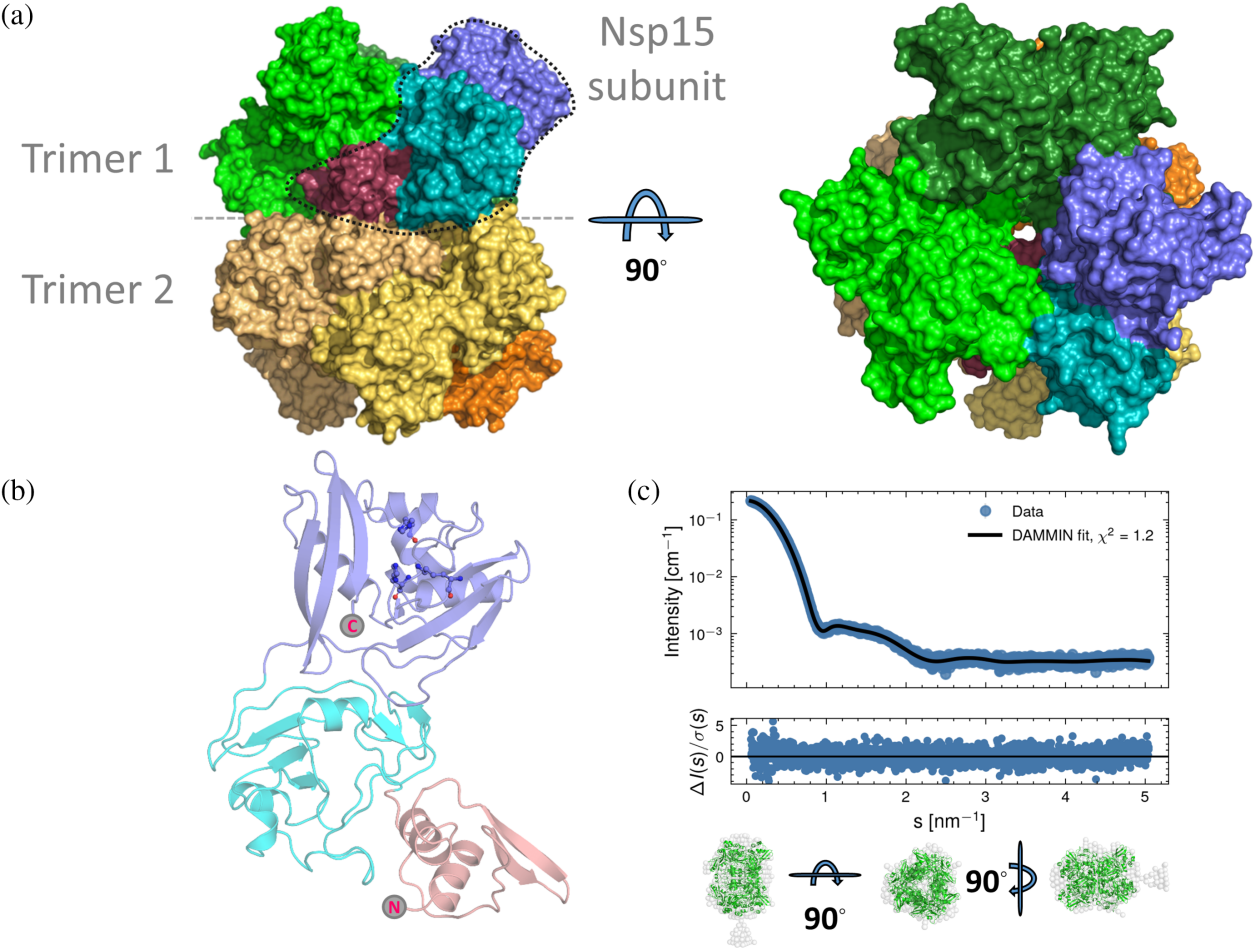
(a) The Nsp15 hexamer in two orientations (rotated 90 degrees). Each Nsp15 subunit has a unique color. For one subunit, circled in dashed line, the three domains of the protein are shown (Nsp15 N‐terminal domain in raspberry, the middle domain in cyan and the EndoU in slate blue). (b) Domain organization and domain boundaries of Nsp15. The catalytic triad is in ball and stick. (c) DAMMIN fit (solid line) and the hexamer model (produced from PDB ID 6wlc, in green) overlaid to the ab‐initio *model (gray beads)* shows that Nsp15 is mostly hexameric in solution as well.

As in all previously reported Nsp15 structures of SARS‐CoV‐2 and other coronaviruses, the Nsp15 subunit is composed of three distinct domains: a N‐terminal domain (a.a. 1–63) which plays an important role in the homo‐oligomerization of Nsp15, a central domain (a.a. 64–181), and a catalytic EndoU domain at the C terminus (a.a. 182–346) (Figure 5a) (Kim et al., 2020; Ricagno et al., 2006; Zhang et al., 2018). Nsp15 is hexameric, formed by a dimer of trimers. The hexameric assembly has an overall surface area of about 80,000 Å^2^ and a 30,000 Å^2^ buried area (Figure 5b). Small‐angle x‐ray scattering measurements showed that the hexameric architecture of the crystal structure fits well with the Nsp15 hexamer in solution (Figure 5c), corroborating that the solution state studies are in line with the x‐ray crystal structure analysis. Small discrepancies between the model and the SAXS curve could be attributed to the N‐terminal His‐tag that was not cleaved off during sample preparation. Ligand‐free Nsp15 showed a slight concentration dependence of the radius of gyration (Figure S5).

For structural analysis of the enzyme–inhibitor complexes, we carried out a crystallographic screening using all confirmed hits from the enzymatic assay. Crystals obtained from co‐crystallization or soaking experiments with the identified inhibitors diffracted at a resolution varying between 1.7 and 3.5 Å. Analysis of these structures resulted in Nsp15–ligand complexes for two inhibitors, that is, the Nsp15‐TAS‐103 structure from co‐crystallization and the Nsp15‐YM‐155 structure from soaking. (Table 2).

**TABLE 2:** X‐ray data collection, phasing, and refinement statistics.

Parameters	Nsp15_TAS‐103 PDB ID:9HH6	Nsp15_YM‐155 PDB ID:9HH5
Data collection		
Wavelength	1.033	1.033
Resolution range^a^	49.26–2.0 (2.03–2.00)	45.14–2.08 (2.12–2.08)
Space group	P 63	P 63
Unit cell (Å, °)	150.49150.49110.09 90 90,120	151.41151.41109.30 90 90,120
Total reflections	444,986 (22637)	491,744 (25066)
Unique reflections	95,339 (4754)	85,181 (4535)
Multiplicity	4.7 (4.8)	5.8 (5.3)
Completeness (%)	99.76 (99.87)	99.69 (99.61)
Mean I/sigma (I)	7.7 (1.6)	12.6 (1.1)
Wilson B‐factor (Å^2^)	41.0	49.12
R‐merge	0.098 (0.868)	0.064 (1.396)
R‐meas (Diederichs & Karplus, 1997)	0.127 (1.121)	0.078 (1.710)
R‐pim (Weiss & Hilgenfeld, 1997)	0.079 (0.699)	0.044 (0.969)
CC1/2 (Karplus & Diederichs, 2012; Karplus & Diederichs, 2015)	0.989 (0.625)	0.999 (0.502)
Refinement		
Reflections used in refinement	95,287 (3174)	95,287 (9508)
Reflections used for R‐free	4755 (166)	4755 (480)
R‐work	0.1616	0.1913
R‐free (Brünger, 1992)	0.1769	0.2053
Number of non‐hydrogen atoms	5977	5222
Macromolecules	5523	4893
Ligands	84	43
Solvent	370	286
Protein residues	692	614
RMS (bonds) (Å)	0.008	0.007
RMS (angles) (°)	1.26	1.12
Ramachandran favored (%)	97.97	97.52
Ramachandran allowed (%)	2.03	2.48
Ramachandran outliers (%)	0	0
Rotamer outliers (%)	0.64	0.72
Clashscore	1.79	0.51
Average B‐factor (Å^2^)	51.41	57.04
Macromolecules	51.35	57.23
Ligands	71.55	69.02
Ligand of interest	82.7 (2 TAS‐103 molecules)	79.1 (1 YM‐155 molecule)
Solvent	47.65	52.01
Number of TLS groups	2	2

^a^ Values in parentheses are for the highest resolution shell.

**FIGURE 6:**
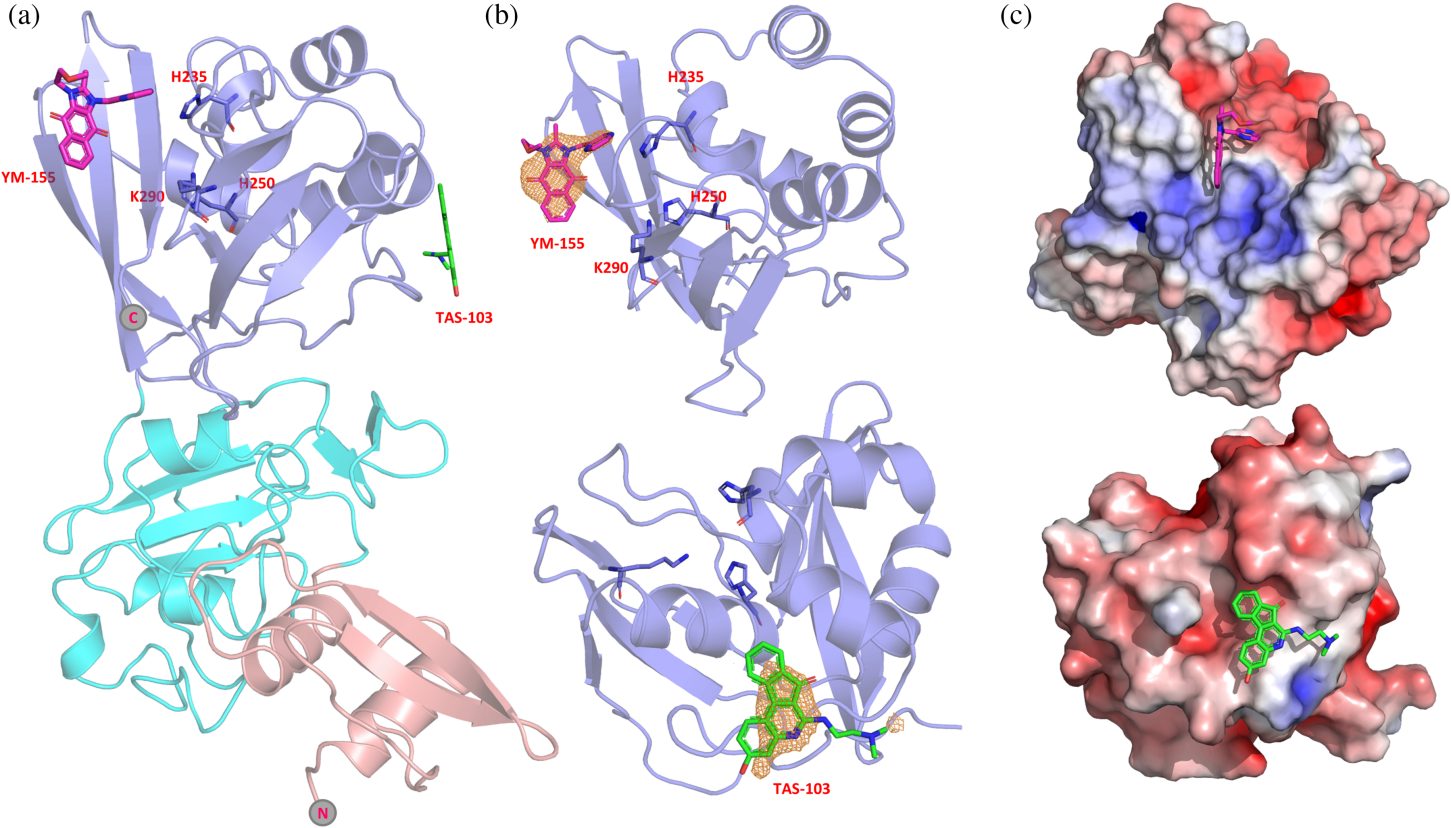
(a) Localization of two identified inhibitors YM‐155 (pink) and TAS‐103 (green) in the catalytic EndoU domain of Nsp15 (for domain color coding see Figure 5). (b) Calculated mFo‐DFc omit map for YM‐155 and TAS‐103 (contoured at a level of 3.0σ for YM‐155 and 2.5σ for TAS‐103, shown as orange mesh). (c) Electrostatic potentials for the catalytic domains of the YM‐155 and TAS‐103 structures.

**FIGURE 7:**
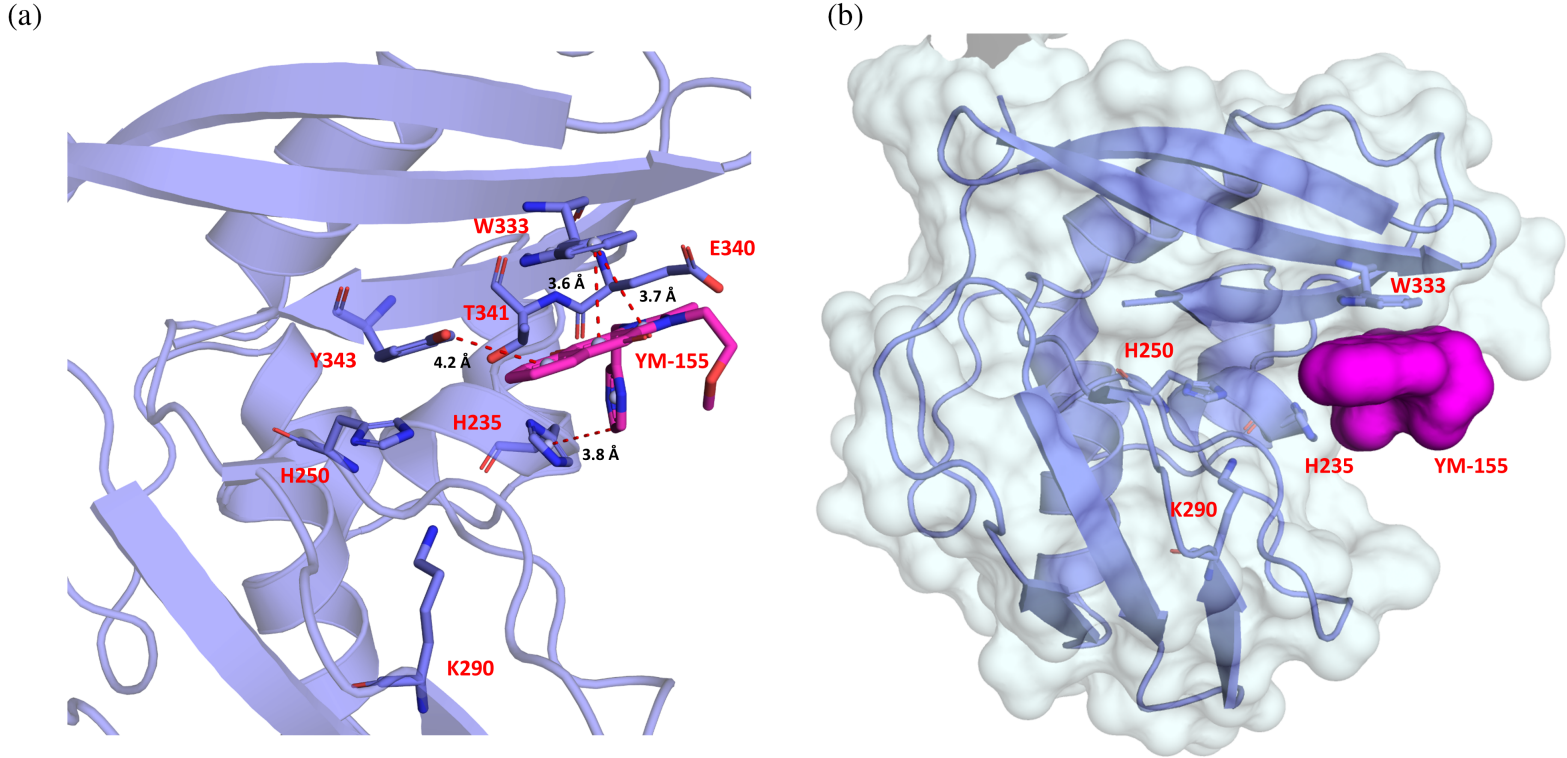
(a) Close up on the interactions of YM‐155 with key residues of the EndoU. (b) The positioning of YM‐155 (in magenta) substantially narrows the uridine binding pocket.

YM‐155 is located next to the active site, forming an interface area of 190 Å^2^ with Nsp15 via van der Waals and π–π stacking interactions (Figures 6 and 7). This interface involves four out of the seven key Nsp15 residues (Kim et al., 2021), i.e., His235, Trp333, Thr341, and Tyr343, and the non‐conserved Glu340.

The ligand binding site of Nsp15 with TAS‐103 is found on the catalytic domain but opposite to the active site, in a cavity that is formed next to the *α*6 and *α*9 helices. The compound interacts with the protein via interactions involving Phe204, Lys205, Pro206, Ile212, Leu215, Glu216, and Lys260, with an interface area of about 240 Å^2^. Next to the TAS‐103 molecule, we observed extra electron density corresponding to at least two additional TAS‐103 molecules, separated by about 3.7 Å, appearing to interact with each other via π‐π stacking. This density is located in the free space created on the tip of the nsp15 hexamer. Although the two additional molecules could be modeled into the structure with lower occupancy, we excluded those from the final model.

The Nsp15 molecules in the two structures reported in this work share very high similarity with the Nsp15 structures deposited in PDB, with an RMSD varying between 0.25 and 0.40 Å and a Q score above 0.97. Even on the sites where the inhibitors are bound, the structural rearrangements are subtle. For the YM‐155 structure, there is a small movement of the Trp333 in comparison with APO Nsp15 structures. A similar effect occurs in other structures with ligands bound at the active center, interacting with this residue. For the TAS‐103 structure, the structural differences with other Nsp15 structures are negligible even at the region where the ligand is bound.

### 2.5 Structural disorder upon soaking experiments

As already mentioned, the Nsp15–inhibitor crystals, obtained either from co‐crystallization or soaking, were isomorphous with the previously reported Nsp15 structures. For a large number of inhibitors though, ~40%, we observed that upon soaking at higher ligand concentrations, there was a distinguishable difference in the c‐axis of the unit cell compared to those reported in PDB structures. In these crystals, the *c*‐axis ranged between 104 and 109 Å upon ligand soaking, compared to the usual length of 110–112 Å. This effect was also present, but not so dramatic, in the YM‐155‐Nsp15 crystal structure, in which the ligand was found only in one of the catalytic domains of the asymmetric unit since the second catalytic domain, especially its active site, could not be fully resolved.

Structural analysis revealed an extensive structural rearrangement and disorder in parts of the catalytic EndoU domain of one of the two subunits (Figure S6). This occurred along the whole catalytic domain and was more pronounced in the area located close to the catalytic triad, that is, residues 200–264, 283–290, 300–319, and 329–345, whereas the other subunit of the asymmetric unit was nearly identical to the ligand‐free structure. Of note is that we observed this phenomenon even in structures in which the soaked inhibitor could not be traced in the electron density.

This disorder, however, did not affect the diffraction ability of the crystals, which diffracted as good as the apo or ligand‐bound Nsp15 crystals, and the disordered crystals had similar data collection statistics to the ones that did not exhibit disorder. Interestingly, a similar phenomenon has been observed in several structures obtained from a fragment screening campaign on Nsp15, and it is suggested to be due to a shift between the active and inactive state (Godoy et al., 2023).

To address whether the addition of the ligand has a similar effect on the Nsp15 structure also in solution, we acquired SAXS data upon the addition of different YM‐155 concentrations (i.e., 1, 5, and 10 mM) (Figure S5b). Upon ligand addition, the protein seemed to be virtually identical to the ligand‐free form. Furthermore, upon addition of the ligand, there is almost no concentration dependency of the radius of gyration for Nsp15.

## 3 MOLECULAR DOCKING STUDY

**FIGURE 8:**
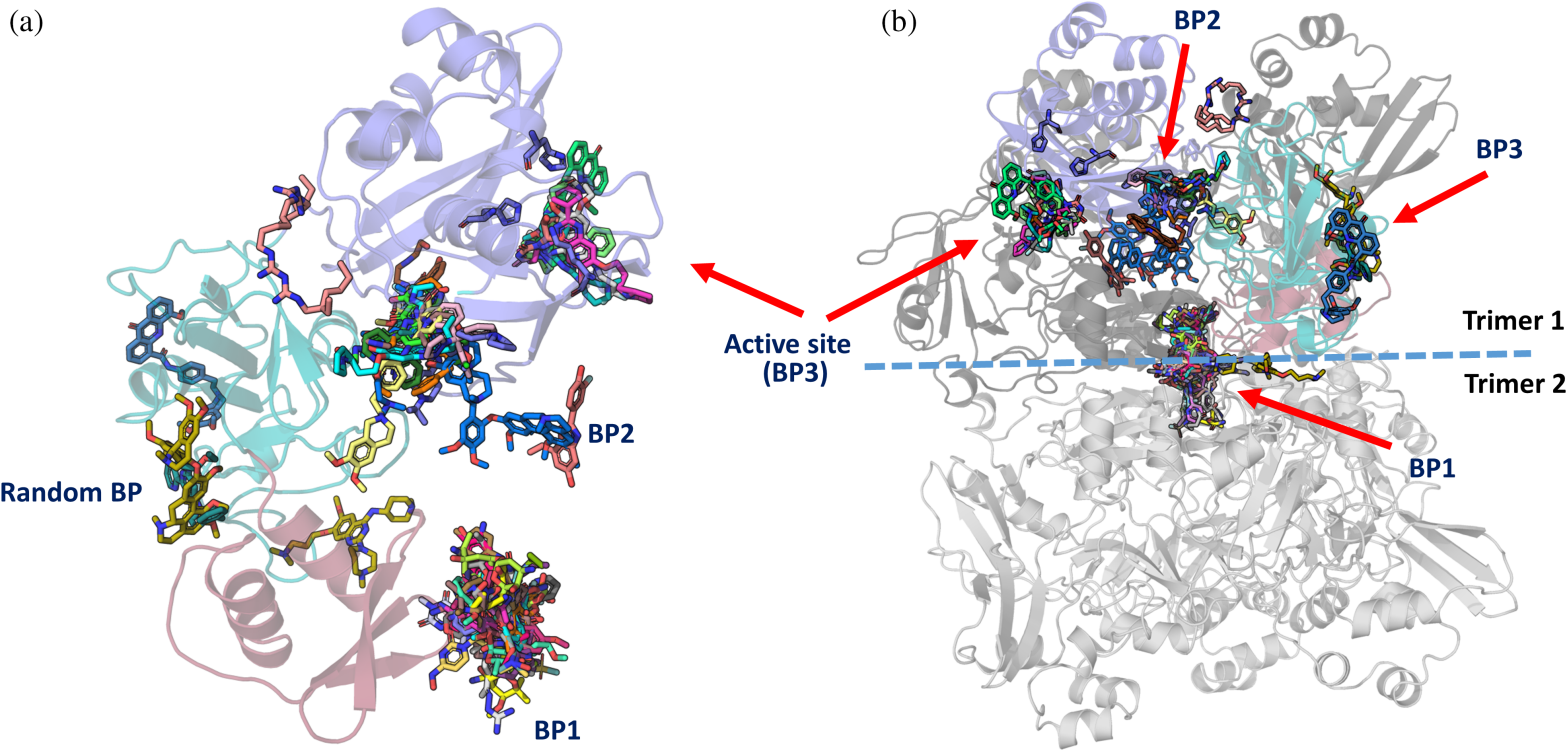
Molecular docking on Nsp15 and the found inhibitors. Identified binding patches in the Nsp15 subunit (a) and in the Nsp15 hexamer (b).

We further conducted a molecular docking study with Nsp15 and 39 inhibitors from our initial screening, together with some already reported compounds bound to Nsp15, for example, tipiracil (Table S2). Since Nsp15 is active as a hexamer, we used the hexameric protein for a global search for ligand binding sites. Most of the inhibitor docking poses appeared to be located at the center of the hexamer and exhibited low binding energy (binding patch 1, (BP1)) (Figure 8). The second binding patch (BP2) is located on the inner part of a subunit of one of the two trimers consisting of the hexameric Nsp15, in a pocket formed mainly by the middle and catalytic domain of one Nsp15 subunit, whereas the third binding patch (BP3) is on the outer surface of the active site domain covering the active site of the Nsp15 subunit (Figure 8 and Table S2). In addition to these three binding patches, we observed a random binding site of nsp15 hexamer, as shown in Figure 8 and Table S2.

The molecular docking study revealed that the 39 chosen compounds for this study show binding at different binding patches (BP1 to BP3), formed by the six subunits (SUs) of the hexameric Nsp15.

In our calculations for the inhibitors for which we did not obtain crystals, UNC0224 (−6.4 kcal/mol; SU4), UNC0638 (−7.1 kcal/mol; SU1), elacridar (−8.9 kcal/mol; SU4), UNC0642 (−7.9 kcal/mol; SU3), UNC0646 (−9.1 kcal/mol; SU3), and GSK J4 (−7.1 kcal/mol; SU3) bind to the active site of nsp15, corroborating our screening results and showing that they could potentially block the active site of the enzyme (Figure S9 and Table S2). The UNC molecules exhibit greater conformational stability and lower energy binding at binding patch 1 (BP1), binding patch 2 (BP2), and the active site (BP3). The GSK J4 compound demonstrates a comparable binding affinity at the active site (BP3) on different subunits of the hexamer; however, one of its binding sites is observed at a random site on the outer surface of the nsp15 hexamer.

The crystallographic analysis of Nsp15 in complex with the found inhibitors revealed YM‐155 to bind next to the active site, whereas TAS‐103 is found on the opposite side of the catalytic domain (Figure 6a). This is in contrast to our global molecular docking study that suggested additional binding of the ligands YM‐155 and TAS‐103 to BP2 and BP1, respectively (Figure 8 and Table S2). To assess whether we can reproduce in silico the crystallographic findings for YM‐155, we performed active site restrained molecular docking. The results showed that YM‐155 can bind to the active site of the Nsp15 with acceptable energy (−6.4 kcal/mol) but in contrast to the crystal structure, the binding takes place right on the active center, as expected for a uridine monophosphate nucleotide, and not next to it, facing the Trp333 as in the crystal structure (Figures 6a, 7a, and 8 and Table S3). These calculations showed that TAS‐103 can also bind to the active site with low binding energy.

## 4 DISCUSSION

### 4.1 In vitro drug repurposing screen

Using a real‐time fluorescence intensity assay, we screened the “Fraunhofer Repurposing Collection,” comprising 5632 approved and investigational drugs. Of the compounds retaining the full inhibition potency in DTT and l‐cysteine buffers, the UNC‐series revealed as active against Nsp15. UNC0224, UNC0631, UNC0646, UNC0638, UNC0642, and UNC0321, possessing a 2,4‐diamino‐6,7‐dimethoxyquinazoline core, have been previously identified as potent and selective G9a/GLP histone methyltransferase inhibitors in a competitive manner with the peptide substrates and non‐competitively with the SAM cofactor (Feoli et al., 2022; Vedadi et al., 2011). We also reported UNC0642 and UNC0646 being active in the cytopathic (CPE) assay against SARS‐CoV‐2 in Vero‐E6 cells with an EC_50_ of 16.73 μM and 35.38 μM, respectively (Zaliani et al., 2022). While UNC0646 has been shown to be cytotoxic with a CC_50_ of 18.62 μM, the CC_50_ of UNC0642 was above the tested concentration range (32 μM). To the best of our knowledge, this compound group has not been reported as SARS‐CoV‐2 Nsp15 inhibitors yet. We also identified IPA‐3, an irreversible inhibitor of the Pak1 kinase (Viaud & Peterson, 2009), previously described as an Nsp15 inhibitor, to be active in our real‐time fluorescence assay (Chen et al., 2023). However, we observed the inhibitory potential to be dependent on the reducing environment. Previously, it was also reported to be active against SARS‐CoV‐2 main protease (Mpro) (Pillaiyar et al., 2022), again depending on the activity, on the reducing assay conditions. Thus, we cannot exclude nonspecific activity on exposed histidine or cysteine residues at the screening concentration of 10 μM.

### 4.2 Structural studies of nsp‐15–ligand complexes

Our molecular docking study suggests the presence of several potential ligand‐binding patches within the Nsp15 hexamer. By molecular docking, we investigated the suitability of these pockets to harbor an Nsp15 inhibitor, showing a series of inhibitors that can potentially bind on the active site; we also found some new putative binding sites. Considering the docking scores with low binding energy and conformational fitting with multiple noncovalent interactions, the central cavity (BP1) appears to be a putative binding site for structurally divergent compounds. Whether a ligand bound to this area can allosterically inhibit the enzyme needs to be further investigated.

Our soaking and co‐crystallization trials using all primary hits resulted in two Nsp15 structures with bound compounds, namely, the YM‐155 and TAS‐103. This rather limited number of compound‐bound Nsp15 structures, reported also in other screening campaigns of Nsp15, where compounds of lower molecular weight were used, is attributed to the high solvent content of the crystals (~75%) (Godoy et al., 2023).

TAS‐103 is an indeno‐quinoline derivative used in cancer research and has been reported to inhibit DNA topoisomerase I/II (Padget et al., 2000). The mode of action for topoisomerase II inhibition has been proposed as a drug‐induced alteration of the nucleic acid substrate topology (Fortune et al., 1999). In contrast, for topoisomerase II alpha, binding of TAS‐103 has been demonstrated, and inhibition by blocking the DNA religation has been proposed (Wilson Byl et al., 1999). TAS‐103 binds to Nsp15 via van der Waals and hydrophobic interactions with residues of helices *α*6 and *α*9 and the loop upstream of *α*6. This area is located at the core of the C‐terminal domain and shapes the entrance of the 10–15 Å wide channel on the two sides of the Nsp15 hexamer. TAS‐103 binding significantly narrows this area. Residues of the α6 helix, such as Glu210, which are highly conserved among coronaviruses, affect the activity of Nsp15 (Guarino et al., 2005). However, it remains to be elucidated how binding of TAS‐103 to Nsp15 could affect the enzyme activity. Due to the loss of activity in l‐cysteine buffer conditions, a non‐specific protein inhibition, for example, by the generation of reactive oxygen species, cannot be fully excluded for this ligand.

For YM‐155, we also observed redox sensitivity in the biochemical assay, suggesting a putative unspecific inhibition of Nsp15. YM‐155 includes a para‐quinone moiety. Previously, this compound class has been analyzed in cancer‐related research for activity on CDC25, a subfamily of dual‐specificity protein tyrosine phosphatases containing a cysteine residue in the active site (Brisson et al., 2005; Zhou et al., 2009). In that study, these compounds were shown to be active only in the presence of DTT and oxygen; thus, the authors assumed them to undergo a reduction to semiquinone anion radicals producing reactive oxygen species that result in irreversible oxidation of cysteine in the active site.

Irrespective of the mechanism of action of YM‐155, the x‐ray crystal structure of Nsp15 in complex with this compound showed inhibitor binding next to the uridine site. This interaction involves the active residue His235, four conserved residues of the EndoU domain (Val315, Trp333, Thr341, and Tyr343), and the non‐conserved Glu340. With the exception of Val315, these residues play an important role for the correct positioning of the ribose on the active site (Tyr343 and to some extent Thr341), the regulation of its activity (Thr341 and Glu340) and the right positioning of the base 3′ at the cleavage site (Trp333), underlying the relevance of this finding (Dahlin et al., 2004; Frazier et al., 2022; Ito et al., 2024; Ortiz‐Alcantara et al., 2010;Wilson Byl et al., 1999; Zhou et al., 2009). The compound mainly interacts with Trp333 via the imidazole and 1–4 benzoquinone rings and with the catalytic residue His235 via the pyrazine ring (Figure 7a). These interactions result in the blockage of the catalytic residue His235 and substantially narrow the uridine binding pocket, making it effectively inaccessible (Figure 7b). Apart from the interaction with His235, which is essential for the enzyme activity, the interaction with Trp333 is also of great importance for the inhibition of Nsp15 since this residue plays a critical role in regulating the stabilization of the base 3' of the cleavage site for dsRNA (Frazier et al., 2022; Ito et al., 2024). For ssRNA, this effect is minimized, whereas for dsRNA, the Trp333Ala mutation results in a 75% reduction of the cleavage in 60 min (Frazier et al., 2022). In nucleotide‐bound structures, Trp333 can form stacking interactions with both purines and pyrimidines; there is a preference though for purines, as shown also in this work, with the purine‐like moiety of YM‐155 (Wilson Byl et al., 1999; Zhou et al., 2009). The interaction of Tyr343 with YM‐155 is also very important since this residue is critical for the uridine specificity, assuring the correct orientation of the ribose within the active site (Bhardwaj et al., 2008; Hackbart et al., 2020; Saramago et al., 2022; Zhang et al., 2018). The same applies for the interactions with Thr341, which is an important residue for the ability of the enzyme to cleave polyU sequences, and for Glu340, the positioning of which plays a role in the regulation of nuclease activity (Frazier et al., 2022; Saramago et al., 2022). A similar type of interaction has been reported in a cryo‐EM structure of Nsp15 bound to a double‐stranded RNA showing that the major groove interacts with residues Trp333, Tyr343, and Glu340 (Frazier et al., 2022). This observation suggests that YM‐155 can hinder both specificity elements of Nsp15, namely, the uridine site at +1 and the purine site at +2, making it an interesting candidate for the synthesis of an Nsp15 inhibitor with lower toxicity than YM‐155 (Godoy et al., 2023).

Apart from this, YM‐155 has already been described as being active against SARS‐CoV‐2 papain‐like protease PLpro and as having an antiviral effect in cell‐based assays (with no cytotoxicity at tested concentrations) (Zhao et al., 2021). The crystal structure of YM‐155 with PLpro has been obtained from soaking experiments and shows one YM‐155 molecule bound to the substrate‐binding pocket, while another one interacts with the thumb domain, and a third compound is bound to the zinc‐finger motif. The protein–ligand interactions are stabilized by intermolecular π–π stacking, hydrophobic interactions, and hydrogen bonds (Hackbart et al., 2020). Due to this finding, we cannot exclude cooperative inhibition of the two SARS‐CoV‐2 targets: Nsp15 and PLpro in cellular models.

## 5 MATERIALS AND METHODS

### 5.1 Expression and purification of Nsp15

N‐ and C‐terminal tagged constructs of NSP15 in the vector PC DNA 3.4 Topo (Thermofisher) were investigated for optimization. Cultures of HEK 293 cells in HEK EXPI 293 medium (Thermofisher) transfected with 1.25 or 1.8 μg/mL vector using 9.0 or 15.5 μg/mL polyethylenimine (PEIMAX 40 kDa, Polysciences) and 10 mM sodium butyrate and 5 mM valproic acid. Cultures were harvested after 48 or 72 h and analyzed by Western blot (His‐Tag). For up‐scaling, 300 mL transfected cultures transfected with 1.6 μg/mL DNA using 9.0 μg/mL PEI in 2 L flasks were used. Cells were aliquoted in 4 pellets per 100 mL medium after treatment with protease inhibitor (Complete, Roche) and stored frozen. The purification protocol included two steps: Ni‐NTA affinity chromatography, followed by size exclusion chromatography using a Superdex 75 10/300 GL column.

The protein expressed in HEK293 cells was provided by Cube Biotech on request and it was used for the establishment of the fluorescent assay and the primary HT screening against the “Fraunhofer Repurposing Collection.” For the hit profiling, the Nsp15–inhibitors complexes biophysical characterization, the SAXS and the crystallographic experiments we have used bacterially expressed Nsp15.

WT‐Nsp15, codon optimized for *E. coli* expression and cloned in the pET‐28‐TEV vector, was expressed in *E. coli* Bl21 (DE3) competent cells using LB medium. Transformed cell cultures were grown to an OD600 of 0.6 to 0.8 prior to their induction with 0.2 mM isopropyl β‐d‐1‐thiogalactopyranoside (IPTG) and incubated overnight at 18°C. Cells were resuspended in lysis buffer supplemented with EDTA‐free protease inhibitor tablets (Roche), DNAse, RNAse, and lysozyme and disrupted using a pneumatic microfluidizer processor. The purification protocol included two steps: Ni‐NTA affinity chromatography, followed by size exclusion chromatography (Superdex 200 increase 10/300 GL, Figure S7 and Table S4).

For the reported work, we used the 6‐His tagged variant of Nsp15 since we were not able to cleave this tag. The intact N‐terminally tagged protein was confirmed by MS analysis (Figure S8).

### 5.2 Primary assay

The Nsp15 endoribonuclease activity is based on the real‐time fluorescence assay described by Kim et al., 2020 (Kim et al., 2020). The single‐stranded modified substrate 6‐Fam‐dArUdAdA‐BHQ‐1 (biomers.net GmbH, Ulm, Germany, # SP10003) contains the Nsp15 specific recognition element uridine. In the absence of enzymatic activity, 6‐carboxyfluorescein (6‐FAM) fluorescence is quenched by the BHQ1 group; upon cleavage, the signal is detected at Ex/Em = 485/535 nm using the PerkinElmer Envision multimode microplate reader. Congo red was used as a positive control for Nsp15 inhibition. In the primary screen, test compounds, positive (50 μM Congo red) and negative (100% DMSO) controls, were transferred to 384‐well assay microplates (Corning® Low Volume 384‐well Black Flat Bottom Polystyrene NBS Microplate, 10 per Bag, without Lid, Non Sterile, Prod. Nr 3820) by acoustic dispensing (Echo® 550R, Labcyte Inc., San José, CA, USA). 5 μL of SARS‐CoV‐2 Nsp15 (Cube Biotech GmbH, Monheim, Germany) were added to compound plates. Plates were sealed and incubated for 30 min at 25°C. After the addition of 5 μ 6‐Fam‐dArUdAdA‐BHQ‐1 substrate, the final concentrations were 1 μM substrate, 1 μM SARS‐CoV‐2 Nsp15, 10 μM compound, and 0.1% DMSO in a total volume of 10 μL/well. The fluorescence signal was measured 15, 30, and 45 min post substrate addition. Inhibition (%) was calculated relative to controls. Results were normalized to 100% inhibition (positive control, Congo red at 50 μM) and 0% inhibition (negative control, DMSO) inhibition. Assay buffer: 20 mM Tris, 5 mM MnCl_2_, 1 mM DTT, 0.01% Tween® 20, pH 7.5. To flag compounds interfering with the assay readout, the enzymatic reaction of Nsp15 with substrate was run to completion, followed by the addition of compounds and detection of the fluorescence signal. Compounds inhibiting the signal were flagged for interference and removed from follow‐up assays.

### 5.3 Crystallization, data collection, and structure determination

The crystals of Nsp15 were grown by vapor diffusion by mixing equal volumes of protein solution (7 mg/mL) and precipitant solution. Seeding was used for all the crystallization attempts.

We crystallized the protein in two different reported conditions (Kim et al., 2021):

1. 0.1 M Tris pH 8.5, 10% w/v Peg 4000, 0.15 mM sodium acetate.
2. 0.1 mM sodium/potassium phosphate pH 6.5, 10% w/v Peg 8000, 0.3 M NaCl.

Crystals were also obtained from previously reported conditions containing citrate ions (Kim et al., 2020), and although they were initially tried in our screening and resulted in high‐resolution datasets, they were not used further because of the presence of the citrate in the Nsp15 catalytic pocket.

Before co‐crystallization of the ligands, the protein (at a concentration of 7 mg/mL) was mixed with the ligand (concentration varying between 2 and 3 mM, depending on the solubility of the ligand) and the mixture was incubated for 1 h at room temperature. For Nsp15–ligand co‐crystals, the cryoprotection solution also included the ligand at the same concentration as in the crystallization drop.

For soaking experiments, we soaked the crystals for an average of 2 h at room temperature. The ligand concentration in the drop varied between 5 and 50 mM, depending on the solubility of the ligand and the solvent in which the ligand was dissolved. For the ligands that were dissolved in DMSO, the highest DMSO concentration in the soaking drop was 20% v/v. For the soaking and harvesting of the crystals, we used the Crystal Shifter (Wright et al., 2021), installed at the user lab of the P11 beamline.

All crystallographic work was conducted at PETRA III at the beamline P11 (Oberthür et al., 2025). Data collection was carried out at 100 K (Teng, 1990), with 25% ethylene glycol as cryo‐protectant, at a wavelength of 1.033 Å, using an Eiger2 X 16 M detector (Donath et al., 2023). Data were processed using XDS (Kabsch, 2010), the solution and initial refinement were done using Dimple (Wojdyr et al., 2013), and the later stages of refinement were performed with Refmac (Murshudov et al., 2011), with twin refinement (for the YM‐155 bound structure: *domain 1*: twin operator H, K, L, and twin fraction 0.86, *domain 2*: twin operator −K, −H, −L, and twin fraction 0.14. For the TAS‐103 bound structure: *domain 1*: twin operator H, K, L, and twin fraction 0.85, *domain 2*: twin operator −K, −H, −L, and twin fraction 0.15) and TLS (Painter & Merritt, 2006). For the TAS‐103 structure, local NCS restraints were used (Usón et al., 1999); for YM‐155, the refinement was performed without NCS restraints. The structure of SARS‐CoV‐2 Nsp15 with PDB id 6WLC was used as a search model (Kim et al., 2021). The Real‐Space Correlation Coefficient (RSCC) and the Real‐Space R‐value (RSR) values for the two inhibitors suggest that the fit to the electron density is good (i.e., RSCC: 0.96 for YM‐155 and 0.93 for TAS‐103 and RSR: 0.09 for YM‐155 and 0.12 for TAS‐103), for the case of the TAS‐103 structure, though the electron density was somehow weaker than expected for the resolution and the overall statistics of this structure (Figure 5). A summary of the data collection and refinement statistics are shown in Table 2.

### 5.4 Small angle x‐ray scattering

SAXS data on Nsp15 were collected at the EMBL beamline P12 (PETRA III, DESY, Hamburg, Germany) using a robotic sample changer and recorded on a Pilatus‐6M pixel detector (Blanchet et al., 2015). Dilution series of Nsp15 were measured at a concentration range of 2, 4, and 8 mg/mL, each with intermittent buffer solution (20 mM HEPES pH 7.5, 150 mM NaCl, and 1 mM TCEP). For the Nsp15 mixed with YM‐155, the protein concentration was 4 mg/mL (~100 μΜ) in all three tested samples, and the ligand concentrations were 1, 5, and 10 mM. After mixing the protein with the ligand, the mixture was briefly spun and incubated at RT for 1 h before measurements. The initial data processing was done using the SASFLOW standard data reduction pipeline at the beamline (Franke et al., 2012). Further data analysis was done by PRIMUS software from the ATSAS 3.2.1 package (Manalastas‐Cantos et al., 2021), using standard procedures and extrapolated to infinite dilution before further processing. The ab initio model was obtained using the DAMMIN software (Svergun, 1999).

### 5.5 nDSF‐biophysical characterization of Nsp15–inhibitor binding

Measurements were performed either using a Prometheus NT.48 fluorimeter (NanoTemper Technologies GmbH, Munich, Germany) at wavelengths of 330 and 350 nm for the titrations, or a Prometheus Panta (NanoTemper Technologies GmbH) for measuring the *T*_m_. DLS measurements were performed using the latter device. The buffer for all measurements consisted of 20 mM HEPES pH 7.5, 150 mM NaCl, 1 mM TCEP, the concentration of Nsp15 was 5 μM and the ligand concentration was 50 μM. After mixing the protein with the ligands, the mixture was briefly spun and incubated at RT for 30 min before loading the capillaries.

Data processing and evaluation was performed using the FoldAffinity web server and Moltenprot web analysis tool (EMBL Hamburg, https://spc.embl-hamburg.de/ and https://spc.embl-hamburg.de/app/moltenprot) (Burastero et al., 2021; Niebling et al., 2021). The temperature range for data fitting was adjusted between 37 and 71°C, and curve fitting was performed using ratio values and “Equilibrium Two State” model.

### 5.6 Molecular docking

To perform a molecular docking study and investigate inhibitor binding site on the Nsp15 protein molecule, we used AutoDockTools 1.5.7 and AutoDock Vina 1 programs (Eberhardt et al., 2021). AutoDock Vina uses the Monte‐Carlo (MC) iterated search combined with the BFGS (Nocedal & Wright, 2006) gradient‐based optimizer (MC/BFGS search algorithm). We used the crystal structure of Nsp15 (pdb id:6wlc) as the receptor molecule since at the time the calculations took place, the refinement of the structures reported in the present study was not finalized. Molecular docking was performed either for the active site region or the whole hexamer. Polar hydrogens were added to the Nsp15 structure using AutoDockTools. 3D structures for compounds to be docked were retrieved from the PubChem database and prepared as well as converted to the pdbqt format using AutoDockTools. Grid boxes describing the search space were defined for molecular docking into the active site and by using the whole hexamer. For all molecular dockings, we used a grid point spacing of 1 Ångstrom. Grid box centered for active site docking: grid points (npts): 44, 38, 28 in *X*, *Y*, and *Z* dimensions; the *X*, *Y*, and *Z* center grid box coordinates were −96.292, 20.168, and −31.749. Number of grid points (npts) grid box centered for the central cavity of hexamer Nsp15: grid points: 110, 106, 116 in *X*, *Y*, and *Z* dimensions; the *X*, *Y*, and *Z* center grid box coordinates were −74.747, 39.832, and 0.248 (Figure S10).

## AUTHOR CONTRIBUTIONS

**Spyros D. Chatziefthymiou:** Conceptualization; investigation; methodology; data curation; validation; writing – original draft; writing – review and editing; visualization; formal analysis. **Maria Kuzikov:** Investigation; conceptualization; methodology; validation; visualization; formal analysis; writing – original draft; writing – review and editing. **Sara Afandi:** Investigation. **Greta Kovacs:** Investigation. **Sukrit Srivastava:** Investigation; visualization; formal analysis; methodology; writing – review and editing. **Andrea Zaliani:** Writing – review and editing. **Andrey Gruzinov:** Writing – review and editing; formal analysis. **Guillaume Pompidor:** Formal analysis; writing – review and editing. **Michele Lunelli:** Formal analysis; writing – review and editing. **Golam Rizvee Ahmed:** Investigation. **Jörg Labahn:** Conceptualization; funding acquisition; project administration; writing – review and editing. **Johanna Hakanpää:** Writing – review and editing; conceptualization; funding acquisition; project administration. **Björn Windshügel:** Funding acquisition; conceptualization; writing – review and editing; project administration; supervision. **Michael Kolbe:** Writing – original draft; writing – review and editing; conceptualization; funding acquisition; project administration; supervision.

## Supporting information


**Data S1.** Supporting Information.

## Data Availability

Data sharing is not applicable to this article as no new data were created or analyzed in this study.
