# Supplementary material for: Identification, validation, and characterization of approved and investigational drugs interfering with the SARS‐CoV‐2 endoribonuclease Nsp15
Source: Protein Sci. 2025 May 15;34(6):e70156. doi: 10.1002/pro.70156 (PMC12079475; doi:10.1002/pro.70156)
Supplement: Supplementary file 1 — Data S1. Supporting Information. [file PRO-34-e70156-s001.docx]

**Table S1.** Characterization of SARS-CoV-2 nsp15 inhibitors identified in the drug repurposing primary screen

| **Inhibitor** | **PS** | **HC** | **IC_50_** | **Inhibition %** | **IC_50_** | **Main peak radius** | **Polydispersity Index (SD)** | **Tm °C** |
| --- | --- | --- | --- | --- | --- | --- | --- | --- |
|  | **Inhibition %** | **inhibition %** | **+DTT** | **+L-Cys** | **+L-Cys** | **DLS** |  | **nDSF**  **(SD)*** |
|  | **10 µM** | **+DTT** | **(µM)** | **10 µM** | **(µM)** | **(SD)** |  |  |
|  |  | **10 µM** |  |  |  |  |  |  |
| Octenidine Dihydrochloride | 98,58 | 92,4 | 3,76 | 102,89 | 4,36 | n.t | n.t | n.t. |
| semapimod | 106,91 | 99,81 | 0,61 | 100,99 | 1,01 | n.t | n.t. | n.t. |
| UNC0646 | 75,26 | 83,16 | 1,36 | 91,29 | 0,73 | 5.32 (0.31) | 0.11 (0.03) | 60.6 (0.1) |
| Oritavancin (diphosphate) | 86,71 | 92,52 | 2,53 | 91,07 | 1,24 | 5.19 (0.14) | 0.28 (0.01) | 60.6 (0.1) |
| Mitoxantrone Hydrochloride | 91,56 | 89,55 | 1,47 | 88,72 | 0,47 | 4.85 () | 0.41 (-) | 60.8 (0.1) |
| UNC0224 | 70,61 | 73,89 | 2,57 | 83,22 | 4 | 5.48 (0.12) | 0.06 (0.01) | 63 (0.1) |
| Epirubicin Hydrochloride | 70,87 | 73,22 | 3,37 | 81,66 | 1,46 | 5.2 (0.21) | 0.11 (0.03) | 60.2 (0.1) |
| UNC0642 | 56,93 | 77,53 | 6,27 | 80,24 | 3,27 | 4.73 (-) | 0.53 (-) | 65.8 (0.7)* |
| UNC0631 | 84,79 | 71,36 | 2,21 | 79,26 | 2,54 | 4.95 (0.17) | 0.18 (0.16) | 60.7 (0.1) |
| GSK J4 HCl | 75,79 | 82,25 | 5,48 | 77,87 | 3,81 | 5.23 (0.17) | 0.08 (0.03) | 60.3 (0.1) |
| Tolonium Chloride | 72,01 | 76,45 | 1,65 | 77,85 | 1,34 | 5.21 (0.03) | 0.24 (0.13) | 59.4 (1.6) |
| Doxorubicin | 80,2 | 65,83 | 3,69 | 74,5 | 2,26 | 5.01 (0.28) | 0.13 (0.08) | 60.2 (0.1) |
| UNC0321 | 63,97 | 77,6 | 3,89 | 72,9 | 3,21 | 5.29 (0.06) | 0.37 (0.01) | 63.0 (0.1) |
| Pixantrone (dimaleate) | 82,33 | 77,48 | 5,23 | 72,81 | 4,37 | 4.96 (0.18) | 0.21 (0.22) | 60.7 (0.1) |
| UNC0638 | 56,7 | 69,5 | 2,84 | 69,89 | 4,77 | 5.23 (0.28) | 0.19 (0.09) | 61.7 (0.2) |
| Propidium-Iodide | 83,07 | 85,94 | 0,95 | 68,98 | 9 | 211,76 | 0.34 (0.21) | 59.9 (0.5) |
| Nemorubicin | 65,2 | 69,25 | 2,61 | 68,98 | 1,3 | 4.94 (0.05) | 0.4 (0.07) | 60.1 (0.3)* |
| Erdafitinib | 57,16 | 66,6 | 3,15 | 68,24 | 4,28 | 5.19 (0.83) | 0.26 (0.3) | 61 (0.2) |
| NSC-663284 | 85,51 | 82,57 | 0,48 | 53,53 | n.t | 4.85 (0.17) | 0.42 (0.16) | 58.8 (0.1) |
| IPA-3 | 86,58 | 89,23 | 0,99 | 51,34 | n.t | failed | failed | 58.6 (0.3) |
| APTO-253 | 61,11 | 78,02 | 7,75 | 48,13 | n.t | 4.48 () | 0.78 (-) | 60.4 (0.1) |
| alphaLapachone | 76,01 | 82,84 | 2,15 | 28,56 | n.t | n.t | n.t | n.t. |
| Delavirdine (mesylate) | 80,73 | 77,28 | 2,29 | 27,24 | n.t | 5.29 (0.32) | 0.1 (0.11) | 60.4 (0.2) |
| Walrycin B | 86,19 | 89,01 | <0.15 | 20,32 | n.t | 5.29 (0.39) | 0.44 (0.08) | 57.4 (0.6) |
| EUK 134 | 70,85 | 56,68 | 3,34 | 16,41 | n.t | 5.34 (0.17) | 0.1 (0.11) | 60.5 (0.2) |
| Elacridar | 68,27 | 72,67 | 4,99 | 15,9 | n.t | 6.05 (0.02) | 0.11 (0.01) | 58.3 (0.3) |
| YM-155 | 74 | 79,74 | 1,23 | 12,37 | n.t | 5.26 (0.2) | 0.1 (0.11) | 60.5 (0.2) |
| BVT-948 | 72,99 | 84,54 | 0,41 | -3,98 | n.t | 5.36 (0.1) | 0.05 (0.04) | 56.9 (0.2) |
| TAS-103 (dihydrochloride) | 63,4 | 63,58 | 6,55 | -33,86 | n.t | 4.94 (0.09) | 0.32 (0.05) | 60.0 (0.1) |

**Table S2.**. Molecular docking study for the hexamer nsp15 protein. The table has listed the ligands showing binds at one of the potential binding patch of six sub units (SU 1 to SU6) from the nsp15 hexamer. Respective binding energy, RMSD* and the binding SU is listed. The binding conformations with lowest binding energy is highlighted in gray. The binding conformation of ligands binding at the active site (Binding site 3) is highlighted in blue. The conformations with random binding to the receptor are excluded. *The RMSD shows the deviation in conformation of the ligand as compared to that of the ligand binding with lowest binding energy with 0 RMSD.

| **Inhibitor** | **Binding Patch 1** | **Binding Patch 2** | **Binding Patch 3**  **(Active Site)** | **Other random binding site of nsp15 hexamer** | |
| --- | --- | --- | --- | --- | --- |
| octenidine | -10.1; 0.000 SU1  -9.7; 2.006 SU1 |  |  |  |  |
| Semapimod | -16.1; 0.000 SU1 |  |  |  |  |
| UNC0646 |  | -9.1; 0.000 SU3 | -8.9; 27.739 SU5 |  |  |
| mitoxantrone |  | -6.1; 37.670 SU6 |  |  | -6.1; 37.863 SU1  -6.0; 39.239 SU1  -7.1; 0.000 SU2  -6.2; 48.513 SU4 |
| UNC0224 |  | -6.8; 0.000 SU2  -6.7; 52.984 SU1 | -6.4; 42.685 SU4 | -6.4; 50.932 SU3 | -6.5; 35.212 SU3 |
| UNC0642 |  | -7.6; 35.792 SU2  -7.5; 34.746 SU5  -7.3; 37.605 SU2  -7.3; 25.539 SU3  -7.3; 22.485 SU3  -7.1; 33.491 SU5  -7.1; 22.723 SU3 | -7.9; 30.939 SU3 |  |  |
| UNC0631 | -13.9; 0.000 SU1  -12.9; 2.852 SU1  -12.1; 2.848 SU1 |  |  |  |  |
| GSK J4 |  | -7.2; 18.726 SU2  -7.1; 39.642 SU5  -7.1; 39.205 SU5  -7.0; 38.084 SU1 | -7.1; 44.407 SU3 |  | -6.7; 29.679 SU2 |
| Tolonium  (Toluidine_Blue) | -10.2; 0.000 SU1  10.1; 2.732 SU1  -10.1; 2.610 SU1  -10.0; 2.849 SU1 |  |  |  |  |
| Doxorubicin | -15.5; 0.000 SU1  -14.5; 2.720 SU1  -14.2; 2.763 SU1 |  |  |  |  |
| UNC0321 | -12.1; 0.000 SU1  -11.7; 2.629 SU1  -11.5; 2.140 SU1 |  |  |  |  |
| Pixantrone | -11.7; 0.000 SU1 |  |  |  |  |
| UNC0638 |  | -7.6; 0.000 SU1  -7.6; 59.788 SU6  -7.3; 1.789 SU1  -7.1; 20.568 SU3  -7.0; 2.395 SU1  -6.9; 58.350 SU4 | -7.1; 21.123 SU1  -6.8; 21.894 SU1 |  |  |
| nemorubicin | -16.1; 0.000 SU1  -13.7; 2.214 SU1  -12.9; 2.515 SU1 |  |  |  |  |
| Erdafitinib | -13.2; 0.000 SU1  -12.8; 2.632 SU1  -12.7; 2.602 SU1 |  |  |  |  |
| NSC663284 | -11.6; 0.000 SU1 |  |  |  |  |
| IPA3 | -12.5; 0.000 SU1  -12.1; 2.077 SU1  -12.1; 2.082 SU1 |  |  |  |  |
| APTO253 |  | -9.4; 0.000 SU2  -9.4; 25.454 SU4  -9.4; 25.371 SU6  -9.3; 52.354 SU1  -9.3; 58.394 SU5  -9.3; 55.479 SU3  -9.0; 61.740 SU5  -8.9; 61.488 SU5 |  |  |  |
| alpha_Lapachone | -12.4; 0.000 SU1  -11.5; 2.451 SU1 |  |  |  |  |
| Delavirdine | -13.5; 0.000 SU1  -13.0; 2.402 SU1  -12.9; 2.263 SU1 |  |  |  |  |
| WalrycinB | -14.0; 0.000 SU1 |  |  |  |  |
| EUK134 | -17.1; 0.000 SU1  -16.2; 1.786 SU1  -16.0; 2.889 SU1  -15.9; 2.541 SU1  -15.4; 2.931 SU1 |  |  |  |  |
| Elacridar |  | -9.4; 0.000 SU1 | -8.9; 65.312 SU4  -8.7; 45.149 SU5 |  | -8.8; 55.023 SU4  -8.7; 51.322 SU5  -9.1; 46.603 SU2 |
| YM155 |  | -7.7; 51.838 SU1  -7.7; 50.664 SU1  -7.5; 46.292 SU3  -7.4; 52.844 SU5  -7.3; 48.125 SU3  -7.0; 47.276 SU3 |  |  | -6.9; 15.740 SU4  -6.9; 36.720 SU2 |
| BVT_948 | -12.1; 0.000 SU1  -11.7; 2.167 SU1  -10.6; 2.327 SU1 | -8.6; 29.268 SU3 |  |  |  |
| TAS-103 | -12.6;0.000 SU1  -12.5;2.257 SU1  -12.1; 2.304 SU1 |  |  |  |  |
|  |  |  |  |  |  |
|  |  |  |  |  |  |
| novobiocin | -14.6; 0.000 SU1 |  |  |  |  |
| Prinaberel | -11.4; 0.000 SU1  -8.7; 2.731 SU1 | -8.3; 23.115 SU6  -7.7; 28.590 SU6 |  |  |  |
| TRAM34 |  | -6.9; 44.962 SU3 |  |  |  |
| Erdafitinib | -13.2; 0.000 SU1  -12.8; 2.632 SU1  -12.7; 2.602 SU1 |  |  |  |  |
| UNC0642 |  | -7.6; 35.792 SU2  -7.5; 34.746 SU5  -7.3; 37.605 SU2  -7.3; 25.539 SU3  -7.3; 22.485 SU3  -7.1; 33.491 SU5  -7.1; 22.723 SU3 | -7.9; 30.939 SU3 |  |  |
| daunorubicin | -15.2; 0.000 SU1  -14.6; 1.935 SU1  -13.4; 2.760 SU1  -12.8; 2.723 SU1 |  |  |  |  |
| ML228 | -15.0; 0.000 SU1  -14.5; 2.623 SU1  -14.2; 1.783 SU1  -14.2; 1.614 SU1  -11.5; 2.439 SU1 |  |  |  |  |
| Leucomethylene | -10.5; 0.000 SU1 | -7.0; 30.013 SU5 |  |  |  |
| Losmapimod | -13.1; 0.000 SU1  -11.9; 2.537 SU1 |  |  |  |  |
| Ethidium Bromide | -13.6; 0.000 SU1  -13.2; 1.294 SU1 |  |  |  |  |
| mitoxantrone |  | -6.1; 37.670 SU6 |  |  | -6.1; 37.863 SU1  -6.0; 39.239 SU1  -7.1; 0.000 SU2  -6.2; 48.513 SU4 |
| Dequalinium | -12.0; 0.000 SU1  -12.0; 1.645 SU1  -11.5; 1.676 SU1 |  |  |  |  |
| Acriflavine | -10.6; 0.000 SU1  -10.5; 0.051 SU1  -10.0; 1.618 SU1 |  |  |  |  |
| Alexidine |  | -6.6; 0.000 SU5  -6.6; 1.277 SU5  -6.4; 3.025 SU5  -6.4; 3.005 SU5  -6.1; 3.528 SU5 |  |  | -6.1; 24.253 SU3 |
| rhodamine123 | -12.8; 0.000 SU1  -12.7; 1.845 SU1 |  |  |  |  |
| Thaliblastine |  | -8.1; 0.000 SU4  -8.1; 47.997 SU1  -8.1; 46.727 SU1  -7.8; 37.658 SU5  -7.7; 17.606 SU2  -8.1; 14.983 SU6 |  |  | -7.9; 36.481 SU2  -8.1; 37.180 SU2  -7.9; 60.290 SU3 |
|  |  |  |  |  |  |
| Tipiracil | -9.4; 0.000 SU1  -9.0; 1.883 SU1  -8.8; 1.970 SU1 |  |  |  |  |

**Table S3.** Active site restrained molecular docking search for the ligands TAS and YM155. The binding conformation of ligands binding at the active site with lowest binding energy is highlighted in blue.

| **Inhibitor** | **Active Site** |
| --- | --- |
| TAS | -7.1; 17.542 |
| YM155 | -7.2; 0.000  -6.6; 3.286  -6.5; 3.290  -6.4; 4.530  -6.4; 1.990  -6.3; 2.749  -6.3; 3.705  -6.1; 6.055 |

**Table S4.** Buffer conditions for the Affinity and Size Exclusion Chromatography purification

| **Affinity Chromatography Buffers** | | | | **SEC buffer** | |
| --- | --- | --- | --- | --- | --- |
|  | **Lysis** | **High-Salt Wash** | **Elution** |  | |
| NaCl | 350 mM | 850 mM | 150 mM | NaCl | 150 mM |
| PBS pH 7.4 | 1x | 1x | 1x | HEPES pH 7.5 | 20 mM |
| TCEP | 1 mM | 1 mM | 1 mM | TCEP | 1 mM |
| Imidazole | 20 mM | 45 mM | 400 mM |  |  |


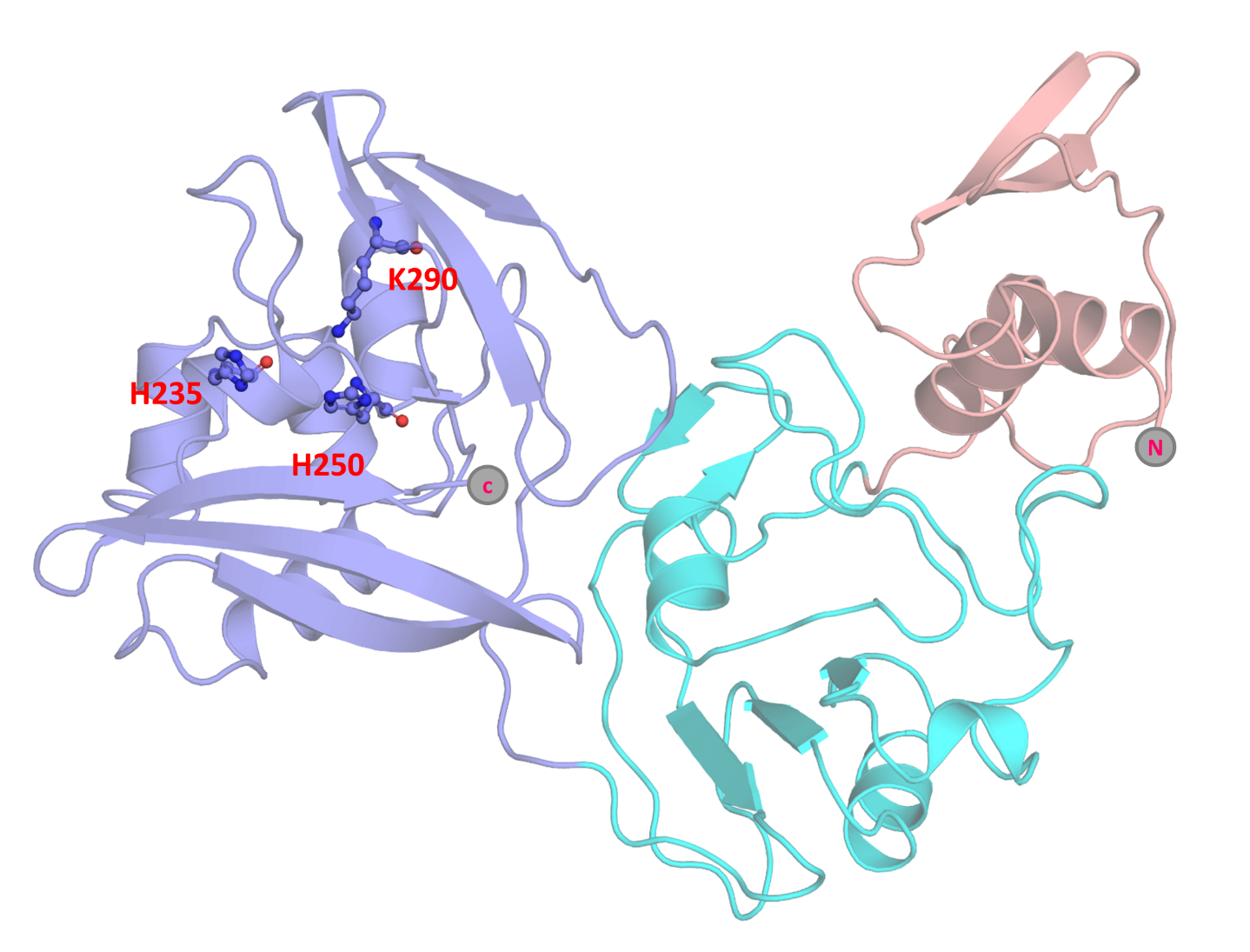


**Fig. S1**. The three domains of Nsp15 (N-terminal domain in red, middle domain in cyan and NendoU catalytic domain in slate). The catalytic residues are shown in stick.


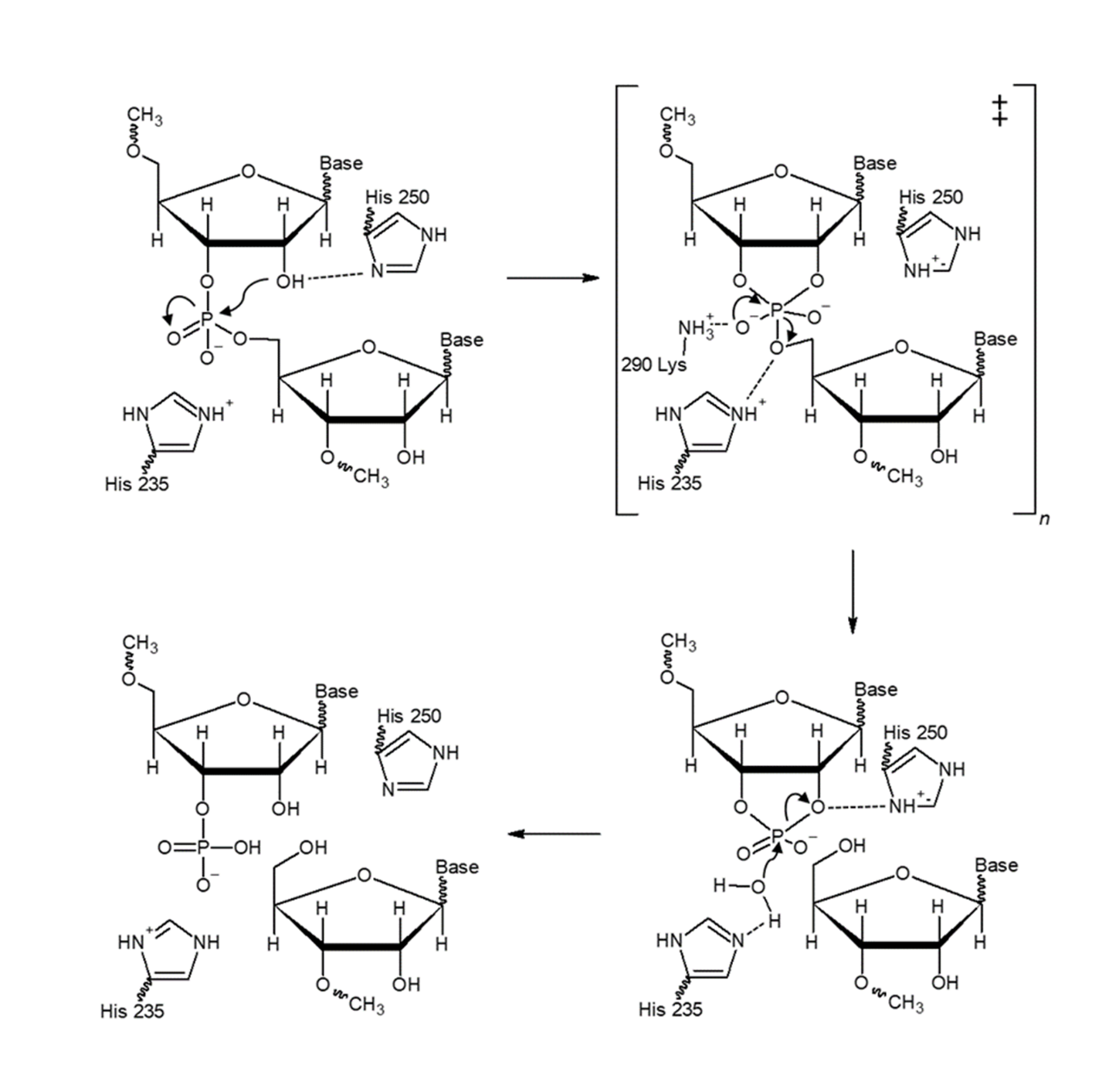


**Fig. S2**. Schematic representation of the Nsp15 proteolytic reaction mechanism


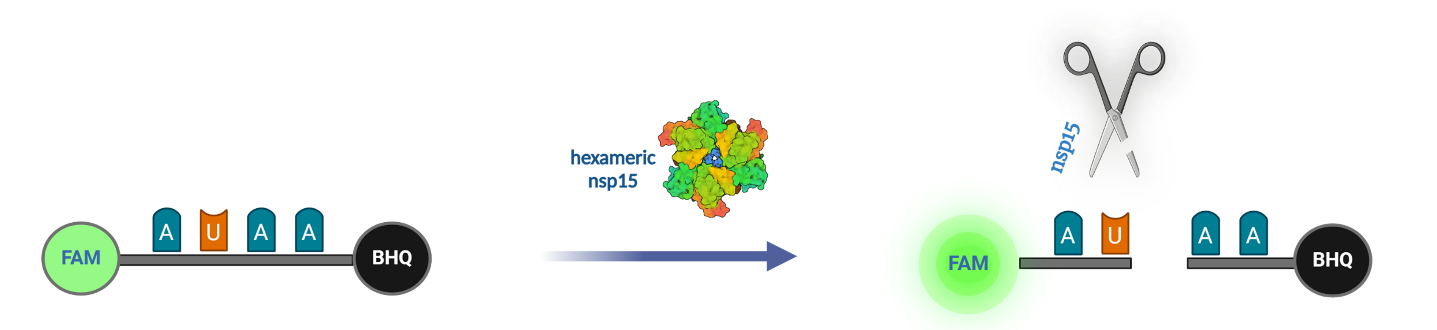


**Fig. S3**. Schematic representation of the real-time fluorescence assay followed in this work. We used a 4-mer oligonucleotide (5′-AUAA) that is cleaved by Nsp15 3′ at the single rU. The fluorescence is quenched by the black hole quencher (BHQ) label in the uncleaved substrate. The enzyme activity is measured by the increase in fluorescence, as the FAM label is released and fluoresces upon cleavage.


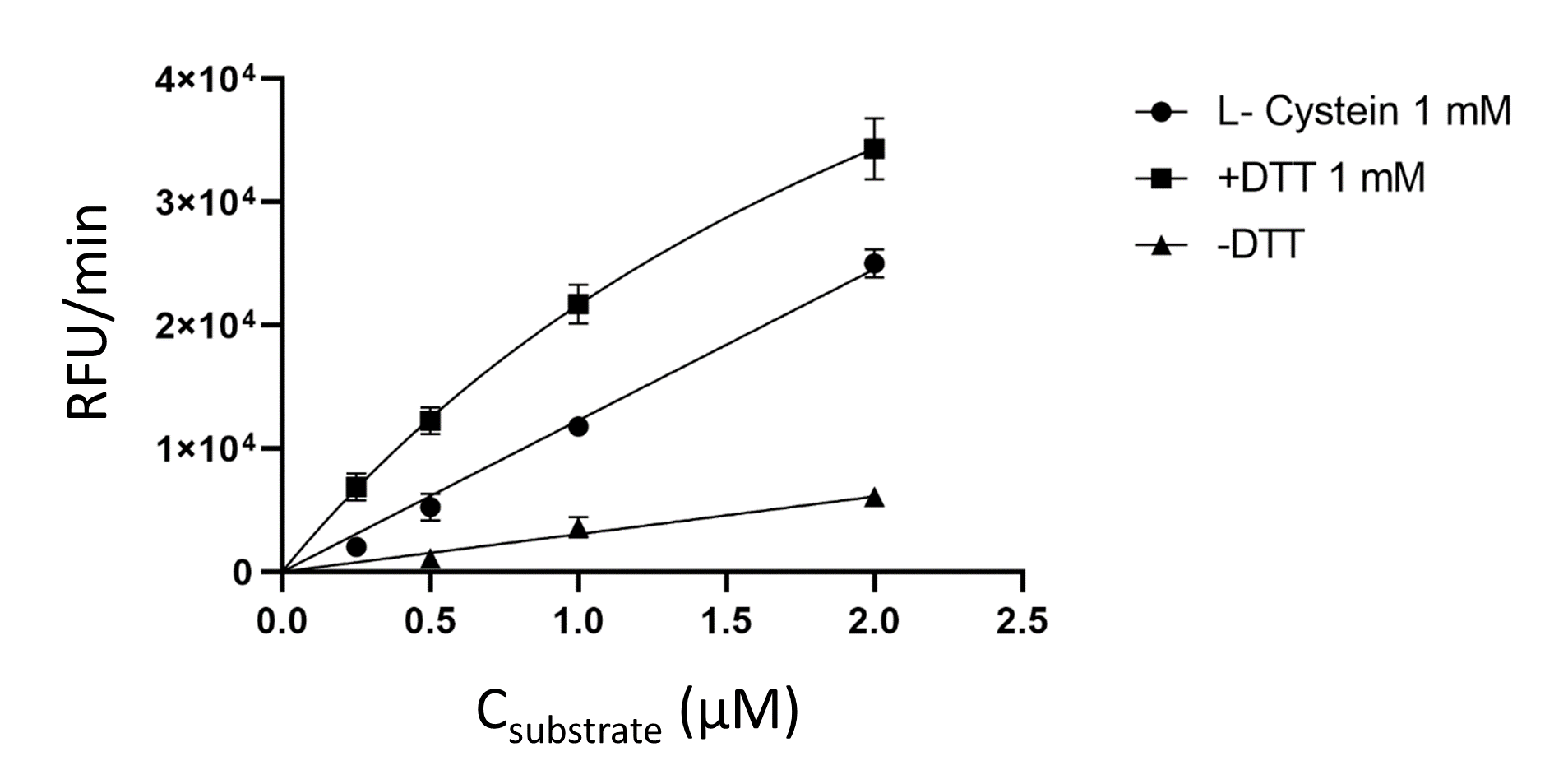


**Fig. S4.** Enzymatic activity in the absence and presence of reducing agents


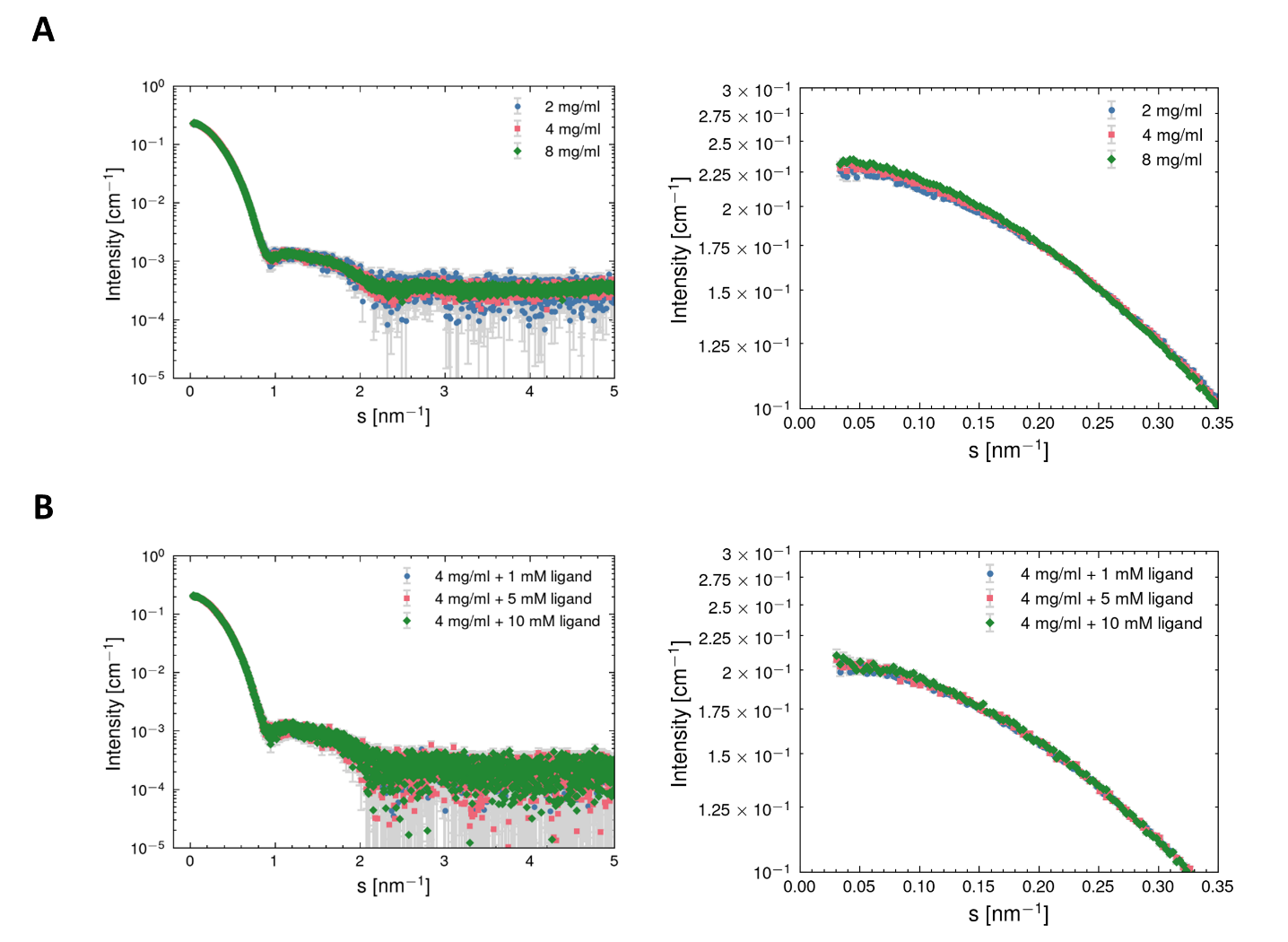


**Fig. S5.** Testing how concentration affects the oligomeric state and the overall structure of APO Nsp15 (**A**) and the Nsp15 in complex with YM-155 (**B**). **A.** Concentration series of Nsp15. The low-angle part of the measurements (shown in the right part of panel A), shows slight concentration dependance of the SAXS data **B.** Concentration series of nsp15 with different YM-155 ligand concentrations. The low-angle part of the measurements (shown in the right part of panel B), shows no concentration dependance.

**
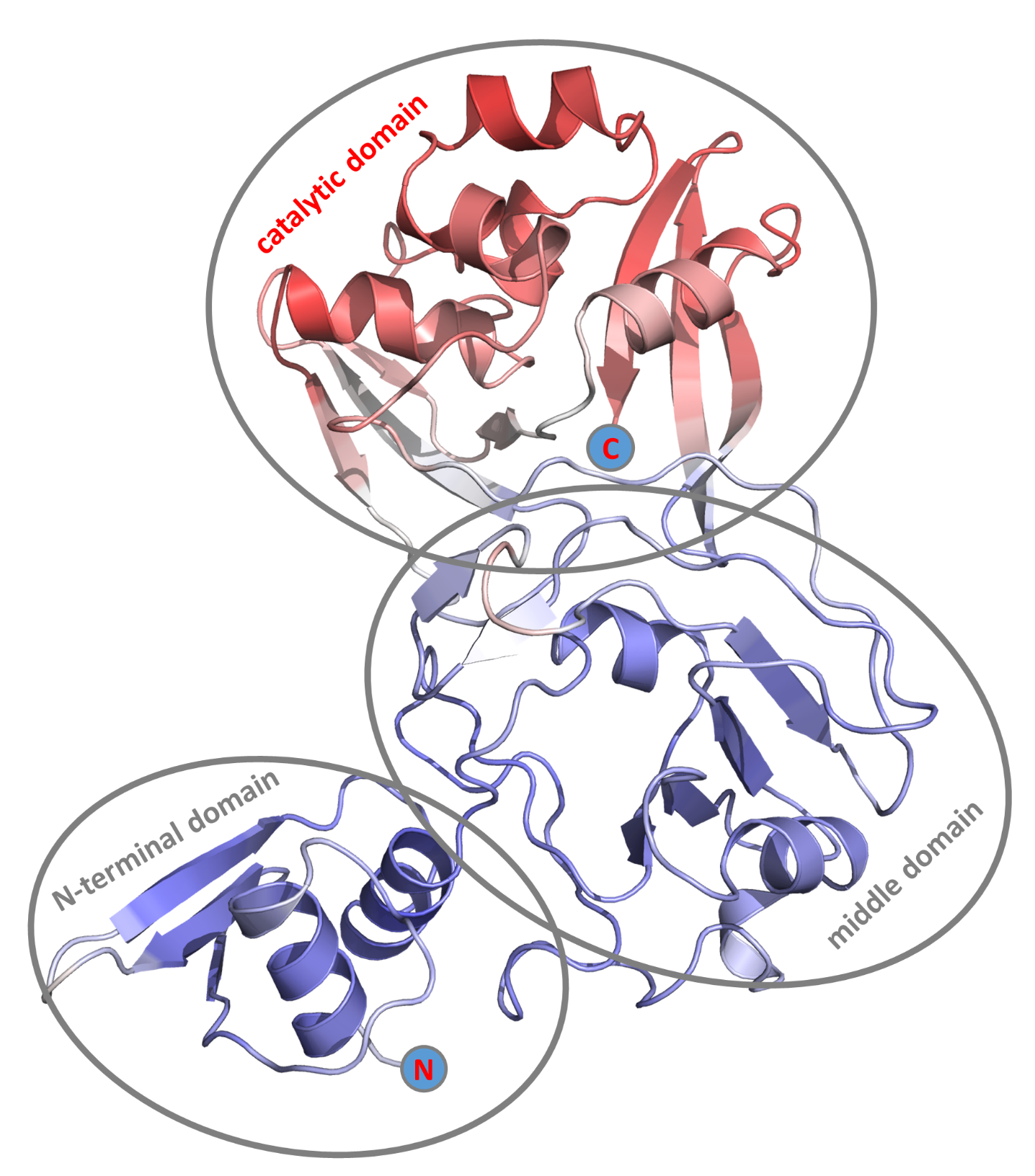
**

**Fig.S6**. Nsp15–YM-155 structure. Disorder of the catalytic domain of the second nsp15 subunit of the asymmetric unit (the disordered regions are colored red, the ordered regions are colored slate).

**
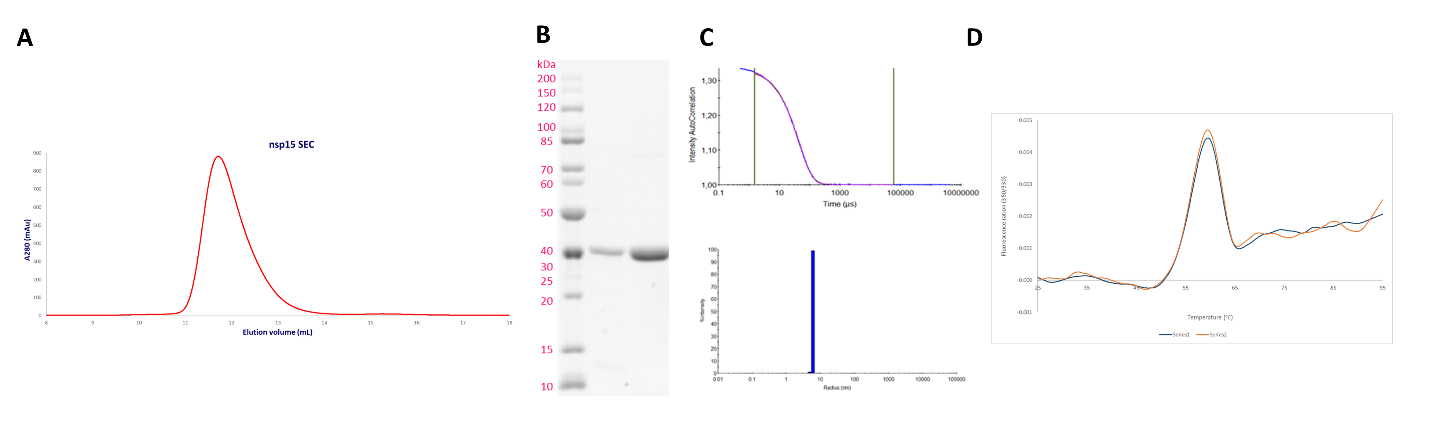
**

**Figure S7.** Biophysical characterization of Nsp15. **A**. Size exclusion chromatography on an analytical Superdex 200 increase 10/300 GL column; calibration standards are indicated. **B**. SDS-PAGE. The molecular weights of some markers near the observed bands are indicated. **C**. DLS verifying the monodispersity of the sample. **D**. nano-DSF on Nsp15 at a concentration of 1mg/mL


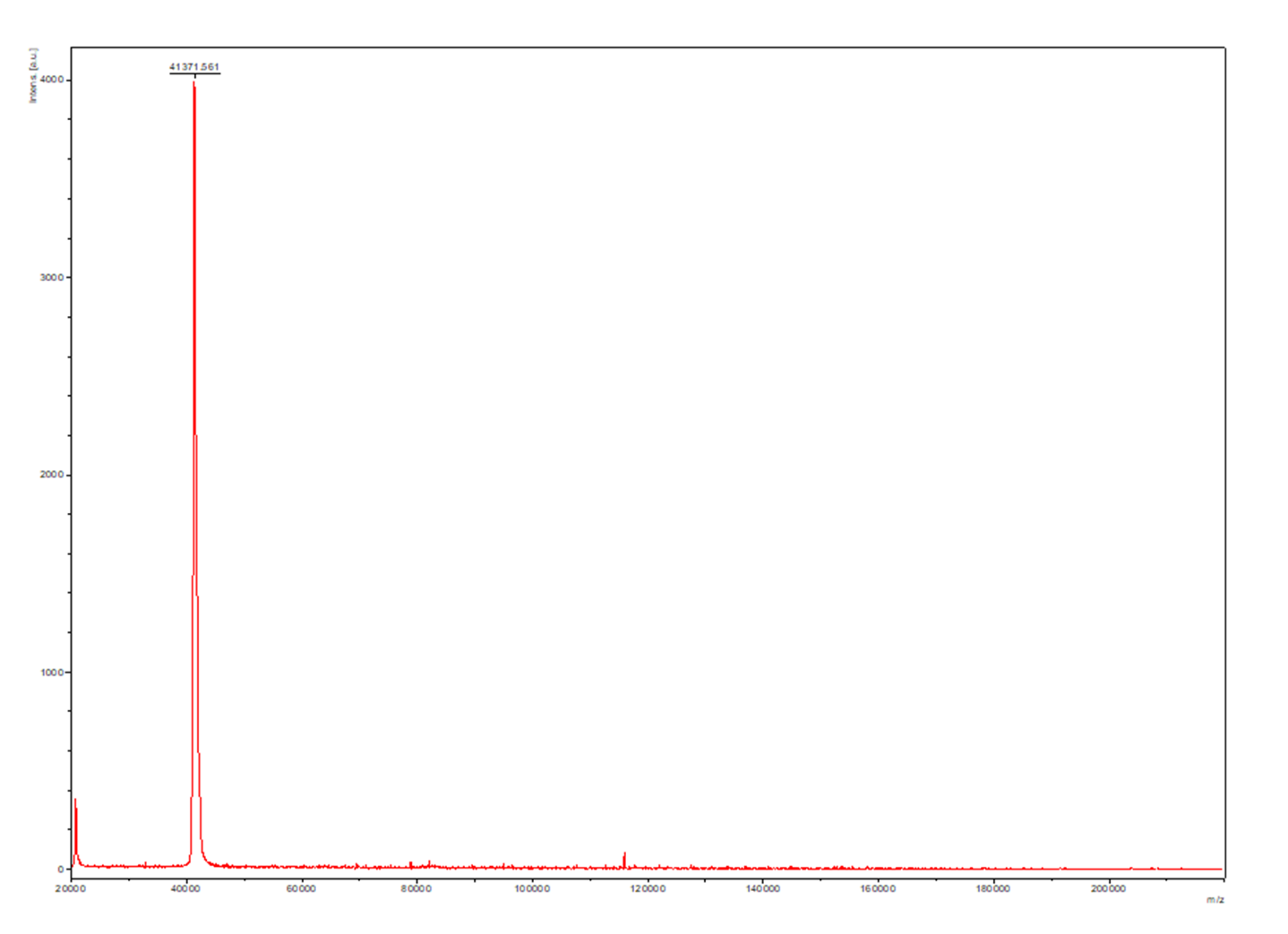


**Fig. S8.** Mass spectrometry on Nsp15 with intact the N-terminal 6His-tag and TEV site


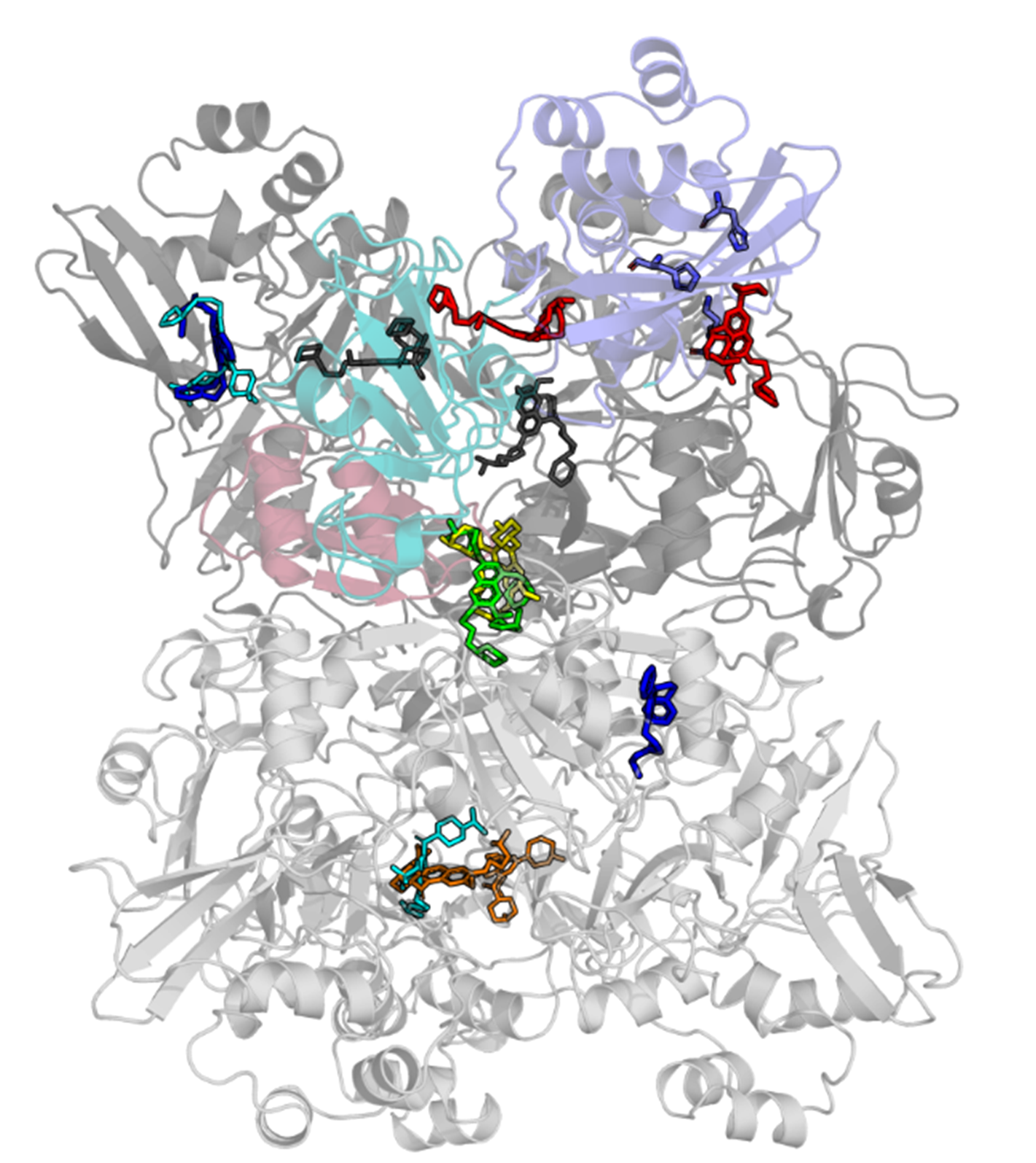


**Fig. S9.** Molecular docking. Distribution of the UNC- and the GSK-J4 ligands along the hexameric Nsp15.

Color code: UNC0628 (red), UNC0631 (green), UNC0321 (yellow), GSK-J4 (blue). UNC0642 (cyan), UNC0224 (orange), UNC0642 (black)


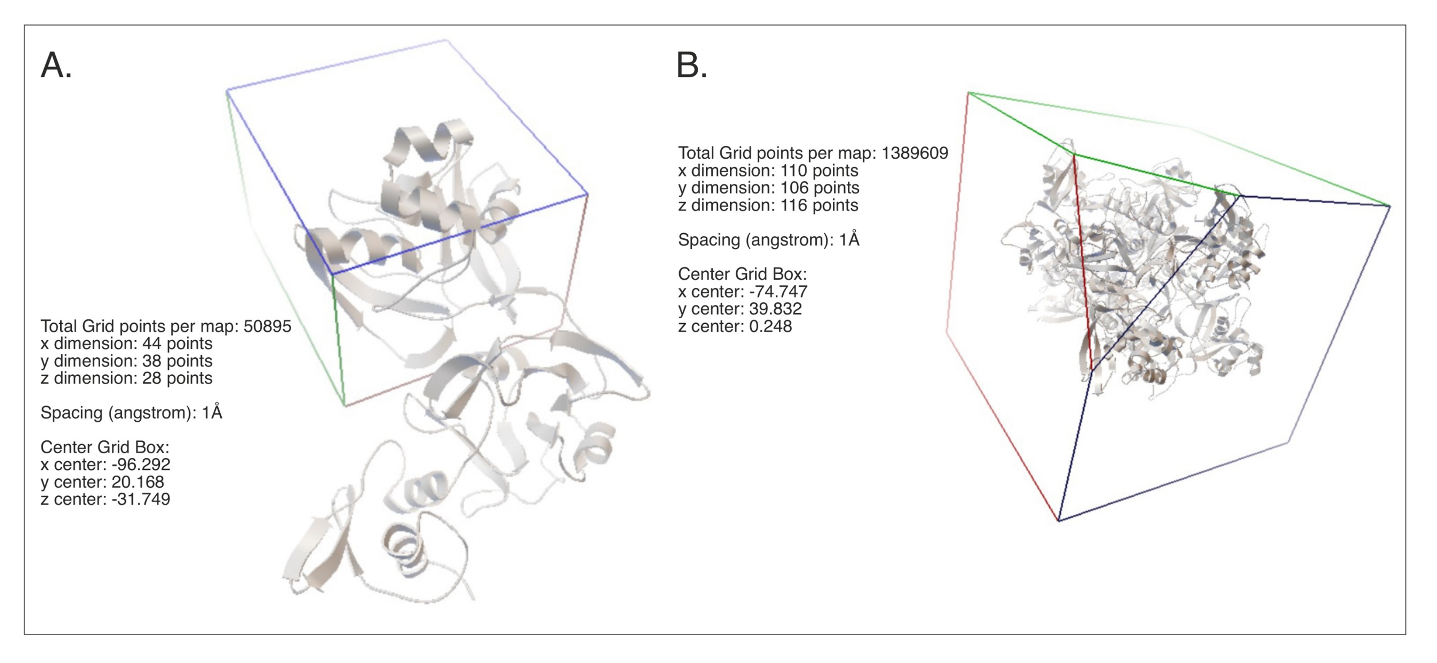


**Fig. S10.** The search space used for the molecular docking. A. Search space covering the active site of nsp15 monomeric unit. B. Search space covering all the six subunits of hexameric nsp15
